# Classical structural identifiability methodology applied to low-dimensional dynamic systems in receptor theory

**DOI:** 10.1007/s10928-023-09870-y

**Published:** 2023-06-30

**Authors:** Carla White, Vivi Rottschäfer, Lloyd Bridge

**Affiliations:** 1 Swansea University, Swansea, UK; 2 Leiden University, Leiden, The Netherlands; 3 University of Amsterdam, Amsterdam, The Netherlands; 4 University of the West of England, Bristol, UK

**Keywords:** Mathematical pharmacology, Receptor theory, Differential equations, Structural identifiability analysis

## Abstract

Mathematical modelling has become a key tool in pharmacological analysis, towards understanding dynamics of cell signalling and quantifying ligand-receptor interactions. Ordinary differential equation (ODE) models in receptor theory may be used to parameterise such interactions using timecourse data, but attention needs to be paid to the theoretical identifiability of the parameters of interest. Identifiability analysis is an often overlooked step in many bio-modelling works. In this paper we introduce structural identifiability analysis (SIA) to the field of receptor theory by applying three classical SIA methods (transfer function, Taylor Series and similarity transformation) to ligand-receptor binding models of biological importance (single ligand and Motulsky-Mahan competition binding at monomers, and a recently presented model of a single ligand binding at receptor dimers). New results are obtained which indicate the identifiable parameters for a single timecourse for Motulsky-Mahan binding and dimerised receptor binding. Importantly, we further consider combinations of experiments which may be performed to overcome issues of non-identifiability, to ensure the practical applicability of the work. The three SIA methods are demonstrated through a tutorial-style approach, using detailed calculations, which show the methods to be tractable for the low-dimensional ODE models.

**Supplementary Information:**

The online version contains supplementary material available at 10.1007/s10928-023-09870-y.

## Introduction

Mathematical models of pharmacological systems have become key in understanding the interactions between ligands and living cells, and as such play a significant role in the development of new therapeutic medicines. These models are often comprised of ordinary differential equations (ODEs) which depend on mechanistic parameters that represent biological processes and whose values are largely unknown [13]. An essential step in using these models requires estimating values of these parameters [52] by fitting to experimental data from, for example, ligand binding assays. Parameter estimation for ODE models of biological systems typically involves optimisation algorithms [17]. However, these fitting routines can result in inaccurate and unreliable estimates [17, 42].

Identifiability analysis is the process of assessing whether it is even theoretically possible to estimate a set of parameters *uniquely* from experimental observations and the dynamic equations [20, 52]. Such analysis is therefore required to determine the reliability of parameter estimates. In particular, structural identifiability analysis (SIA) uses the model structure, together with observed outputs, to determine whether parameters can be returned successfully, given perfect, noiseless and bias-free observations [3, 51].

### A simple demonstration of the issue of (non-) identifiability

**Fig. 1:**
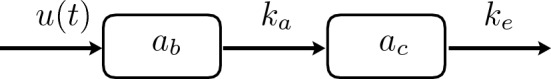
A two-compartment PK model for oral absorption of a drug

Theory and methodology for identifiability analysis have been developed and considered within the context of compartmental models for pharmacokinetics (PK) [4, 14, 22, 23, 29, 30]. We use a simple PK model to demonstrate the issue of non-identifiability. Consider the schematic shown in Fig. 1, which depicts a two-compartment model for oral absorption of a drug. In response to a drug input rate *u*(*t*) (eg. a bolus dose), the drug amount in the *absorption compartment* is $a_{b}(t)$ and the drug amount in the *central compartment* is $a_{c}(t)$.

Assuming linear pharmacokinetics gives the following linear system of ordinary differential equations (ODEs) for $a_{b}(t)$ and $a_{c}(t)$:

1a $$\begin{aligned} \frac{da_{b}}{dt}&= -k_{a}a_{b} + u(t), \end{aligned}$$

1b $$\begin{aligned} \frac{da_{c}}{dt}&= k_{a}a_{b} -k_{e} a_{c}, \end{aligned}$$

1c $$\begin{aligned} a_{b}(0)&=a_{c}(0)=0, \end{aligned}$$

where *u*(*t*) is the input dosing rate. It is straightforward to show [22, 27] that the drug level in the absorption compartment following a single bolus dose $D_{0}$ (so that $u(t)=FD_{0}\delta (t)$, where *F* is the bioavailability factor [22, 27, 31] and $\delta$ is the Dirac delta function) is given by

$$\begin{aligned} a_{b}(t)=D_{0}Fe^{-k_{a}t}. \end{aligned}$$

Note that an equivalent problem is given by setting $u(t)\equiv 0$ and setting $a_{b}(0)=FD_{0}$. The drug level in the central compartment is found to be

2 $$\begin{aligned} a_{c}(t)=\frac{D_{0}F k_{a}}{k_{a}-k_{e}}\left( e^{-k_{e}t}-e^{-k_{a}t}\right) . \end{aligned}$$

**Fig. 2:**
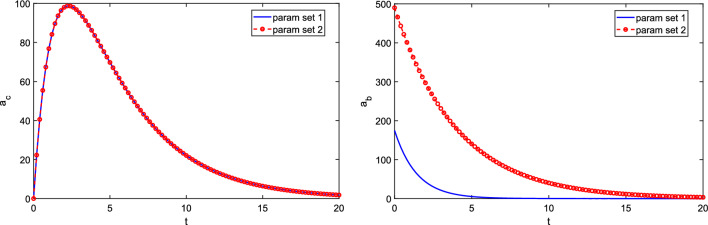
Time courses for observed output $a_{c}(t)$ and non-observed $a_{b}(t)$ for the two-compartment PK model (1a–c), for the two parameter sets given in Table 1

In PK studies using such models, $a_{c}(t)$ (or some scaled drug amount, for example, drug concentration given by $a_{c}/V$ where *V* is the volume of distribution [27]) would be of primary interest and would be the measured/observed output of the ODE system. Suppose a dose $D_{0}=500$ (in arbitrary units) is given at time $t=0$. In Fig. 2, we show $a_{c}(t)$ and $a_{b}(t)$ for the two different sets of parameter values ($k_{a}$, $k_{e}$ and *F*) given in Table 1. Despite the different parameter values, the two parameter sets give identical observed output $a_{c}(t)$ (despite the unobserved, intermediate timecourses for $a_{b}(t)$ being different). Hence the three parameters $k_{a}$, $k_{e}$ and *F* are theoretically unidentifiable from such a time course; their values cannot be determined without further *a priori* knowledge. This system in fact gives an example of *local identifiability*, where multiple distinct values, unique in a neighbourhood, solve the problem - see definitions, e.g., in [12]. Clearly, issues of identifiability should be considered as part of any parameter estimation implementation using real data.

**Table 1:** Parameter values for PK model (2)

	$k_{a}$	$k_{e}$	*F*
Parameter set 1	0.7	0.25	0.35
Parameter set 2	0.25	0.7	0.98

### Classical structural identifiability analysis (SIA) methods

The origins of SIA lie in the works of Kalman [33] for linear ODE systems, and Hermann and Krener [25] for nonlinear models. Since these works, many methods have emerged to assess the identifiability of a given model. For linear models, the Laplace transform (transfer function) method [4] may be used. For linear or nonlinear models, Taylor series expansions [45] and similarity transforms [8, 28] are applicable. In theory, the methodology underlying these three approaches applies to an *n*-dimensional ODE system. In practice, the complexity of the algebraic manipulation involved in the required computations may limit their applicability to low-dimensional systems. Often compartment models arising in pharmacokinetics are used as suitable low-dimensional examples of the application of SIA methods [10, 21, 50].

SIA methodology has grown as a field, and a number of methods and software packages have been developed, which are particularly useful for nonlinear or high-dimensional systems, many making use of symbolic algebra [2, 5, 12, 13, 37–39]. More recent SIA algorithms include singular value decomposition of sensitivity matrices [32, 48] and scaling approaches [7]. While the field of SIA has grown, the difficulties in applying both classical and newly developed methods in general are still noted throughout the literature. The intractability of the required algebraic manipulations for large and/or nonlinear ODE systems has proven to be a “persistent bottle-neck” [48] and most of the available methods may be “too complex mathematically for the general practitioner” [7]. It is this limitation of SIA methods that has resulted in only a very small proportion of theoretical biology studies considering the identifiability issue [7].

Receptor theory is a core component of pharmacological analysis which considers the interactions between ligands and receptors, and the implications of these interactions, at the top of signal transduction pathways of pharmacological interest. A key aim of analytical pharmacology is to use timecourse data to estimate kinetic association and dissociation parameters to quantify ligand-receptor interactions. Similar to in many systems biology modelling work, SIA is frequently overlooked for ligand binding models. The aim of the current work is an informative tutorial which brings classical SIA methods to the field of receptor theory, in particular considering the dynamics of ligand binding models of importance.

SIA has been applied to pharmacodynamics models with drug *effect* as the observed output [31], and to more complex PK/effect models with measured *outputs beyond/downstream of ligand binding* [11, 16]. Ligand binding has been considered in a PK context with total ligand (free ligand plus bound ligand) as the output [9]. Identifiability for *equilibrium* ligand binding is only considered in detail in [42, 43]. Here we follow a receptor theory approach and consider SIA for models of ligand binding assay *dynamics* with total *ligand-bound receptor* as the readout for binding scenarios of practical importance [34, 44, 55]. Given the similar size and structure of many ligand binding receptor theory ODE models [34, 36, 54] to the PK models for which the three “classical” SIA methods have been demonstrated, we propose that application of these classical methods to widely used receptor theory models is valuable on two fronts. Firstly, the analysis will provide new results on the identifiability of key kinetic parameters. Secondly, the study will bring the SIA methodology to a new audience in receptor theory.

### Paper overview

Here we consider three ligand-binding scenarios. The first concerns a single ligand type binding monomeric receptors, which is the starting point for much of receptor theory [34, 35]. The second scenario is the widely used competition binding model of Motulsky-Mahan [44], wherein two different ligand types compete for monomeric receptors. This model can be formulated as a second order linear ODE system with four kinetic rate constants and the total receptor concentration as the “unknown” parameters. It is known intuitively that a subset of the parameters may be estimated if the other parameters are already known [44], but formal analysis of what is theoretically identifiable from a single experiment has not been presented. Furthermore, due to its widespread use, the practicality of parameter estimation for this model continues to receive attention [15, 18, 49]. Theoretical (SIA) questions still remain.

The third scenario we consider is that of a single type of ligand binding to homo-dimerised G protein-coupled receptors (GPCRs). This model is a natural extension of the monomeric receptor model to account for the growing acceptance of the existence of GPCR dimers. This model may also be formulated as a second order linear ODE system. An analysis of the binding kinetics appears in [55], but SIA questions have not yet been addressed.

The remainder of this paper is organised as follows. In Sect. 2, we introduce the three “classical” methods by their application to the simple model for ligand binding to monomeric receptor. In Sect. 3, we apply the three classical methods to the Motulsky-Mahan and GPCR dimer models, yielding new identifiability results of practical importance to the pharma-modelling community, and also contrasting the three approaches. In Sect. 4, we extend the analysis to consider further experiments (including equilibrium binding, washout and multiple timecourses) which can be performed to overcome issues of non-identifiability. We conclude in Sect. 5 with a discussion of our main results and contribution to the literature.

## Methods: applying SIA to monomeric receptor binding model

To demonstrate the methods, we use a simple model of a monomeric receptor binding with a ligand. While the analysis of this model is mostly trivial, it will allow us to explain the process of using each method to determine identifiability. Furthermore, this model has been the basis of much of the theoretical foundations of receptor theory of the past few decades and is still widely used in drug development research [34, 35].

We assume that a ligand, *A*, binds to the monomeric receptor *R* with association and dissociation rates $k_{a+}$ and $k_{a-}$ respectively, to create the complex *AR*. This can be described as a chemical reaction as:

$${\text{A}}\, + \,{\text{R}}\,\underset{{k_{{a - }} }}{\overset{{k_{{a + }} }}{\rightleftharpoons}}\,{\text{AR}}$$

The law of mass action, assuming $[A]$ is held constant, gives the linear system of ODEs Now, asssuming that the only known value is

3a $$[R]^{\prime}= -k_{a+}[A][R]+ k_{a-}[AR],$$

3b $$[AR]^{\prime} = k_{{a + }} [A][R] - k_{{a - }} [AR].$$

These ODEs, together with the initial conditions

3c $$\begin{aligned}{}[R](0)=R_{tot},\qquad [AR](0)=0, \end{aligned}$$

form the initial value problem describing the kinetics of the system, where $R_{tot}$ is the total receptor concentration. Throughout our work, we focus on constant ligand concentration, linear models, in line with typical, classical analyses in the literature ([34, 44]). We note that the less-common assumption of of significant ligand depletion effects would yield a nonlinear system for which the transfer function method (see below) is not applicable.

For the model 3, the concentration of $[AR]$ is measured experimentally at a number of timepoints, hence the *observed output* is

3d $$\begin{aligned} y=[AR]. \end{aligned}$$

Our assumption here uses total bound ligand as a direct readout (e.g. [44, 55]). More detailed models for specific binding assays could consider a further constant of proportionality or Hill function measured response [6, 34].

Now, asssuming that the only known value is the ligand concentration $[A]$, the vector of unknown parameters in the initial value problem (3a–d) is given by ${\textbf {p}}=(k_{a+},k_{a-},R_{tot})$.

For this simple model, it is possible to solve the initial value problem and obtain exact solutions. The measured output solution is found to be

4 $$\begin{aligned}{}[AR](t)=\frac{k_{a+}[A]R_{tot}}{k_{a+}[A]+k_{a-}}\left( 1-e^{-(k_{a+}[A]+k_{a-})t}\right) . \end{aligned}$$

From this we can see that there are two identifiable parameter combinations, namely

5 $$\begin{aligned} \frac{k_{a+}[A]R_{tot}}{k_{a+}[A]+k_{a-}}\qquad \text {and}\qquad k_{a+}[A]+k_{a-}. \end{aligned}$$

Noting that the second of these expressions appears in the denominator of the first, we see that the identifiable parameter combinations may be simplified and listed as

6 $$\begin{aligned} k_{a+}[A]R_{tot}\qquad \text {and}\qquad k_{a+}[A]+k_{a-}. \end{aligned}$$

We would therefore expect identifiability methods to also find these two identifiable parameter combinations. In particular, we note that a single experiment does not show the primary parameters of interest, $k_{a+}$ and $k_{a-}$, as identifiable.

### The transfer function method

The first method we apply is the transfer function method, which makes use of the Laplace transform. This method was first proposed by Bellman and Åström [4] and is one that is simple in nature, but restricted to linear time-invariant systems.

Before applying this method to the monomer-ligand binding model, we first note that, the only input to the system is captured by the initial conditions (3c). We choose to reformulate in such a way that the ligand input is captured by a forcing term in the ODE, as in similar analysis in PK and control theory [1]. We use a conservation law to reduce the system dimensions, in line with our previous work [55]. In (3a–d), total receptor is conserved such that

7 $$\begin{aligned} R_{tot}=[R]+[AR], \end{aligned}$$

which we use to reduce the system by eliminating $[R]$. Upon substitution, we find the single differential equation

8a $$\begin{aligned} \frac{d[AR]}{dt}&=-(k_{a+}[A]+k_{a-})[AR]+ k_{a+}[A]R_{tot}, \end{aligned}$$

with initial condition

8b $$\begin{aligned}{}[AR](0)=0, \end{aligned}$$

and output

8c $$\begin{aligned} y=[AR]. \end{aligned}$$

The system is now in the format

9 $$\begin{aligned} \sum ({\textbf {p}})=\begin{bmatrix} {\textbf {x}}'=F{\textbf {x}}+G{\textbf {u}} \\ {\textbf {y}}=H{\textbf {x}} \\ {\textbf {x}}(0)={\textbf {x}}_0 \end{bmatrix} \end{aligned},$$

with

10 $$\begin{aligned} {\textbf {x}}&=[AR],\qquad F=-(k_{a+}[A]+k_{a-}),\qquad G=k_{a+}[A]R_{tot}, \nonumber \\ H&=1,\qquad {\textbf {u}}=1, \qquad {\textbf {x}}_{0}=0. \end{aligned}$$

The transfer function describing the input–output relation for the system $\sum$ in (9) (see [1]), is

11 $$\begin{aligned} Q(s)=H(sI-F)^{-1}G, \end{aligned}$$

where *I* is the $n\times n$ identity matrix if ${\textbf {x}}$ is an *n*-vector. For the model given by (10), we find that

12 $$\begin{aligned} Q(s)=H(sI-F)^{-1}G=\frac{k_{a+}[A]R_{tot}}{s+(k_{a+}[A]+k_{a-})}. \end{aligned}$$

The parameter-dependent coefficients of powers of *s* (including the $s^0$ terms) in both the numerator and denominator of *Q*(*s*), give the uniquely identifiable parameter combinations. We find a vector of parameter combinations that are identifiable to be

13 $$\begin{aligned} \varvec{\zeta }({\textbf {p}})=\begin{bmatrix} k_{a+}[A]R_{tot}\\ k_{a+}[A]+k_{a-}\end{bmatrix}. \end{aligned}$$

To determine identifiability we assume the existence of an alternate vector of parameters which would yield the same output as the original parameters ${\textbf {p}}=(k_{a+},k_{a-},R_{tot})$. Denote this alternative vector by $\widetilde{{\textbf {p}}}=(\widetilde{k_{a+}},\widetilde{k_{a-}},\widetilde{R_{tot}})$. Then a second vector of parameter combinations is $\varvec{\zeta }(\widetilde{{\textbf {p}}})$. Now we set $\varvec{\zeta }({\textbf {p}})=\varvec{\zeta }(\widetilde{{\textbf {p}}})$ and solve for ${\textbf {p}}$. Each parameter, $p_i$, is deemed *structurally globally identifiable* if, in solving this system of equations, it is found that $p_i=\widetilde{p_i}$. Similarly, the parameter is *structurally locally identifiable* if there is a fixed number of possibilities for $p_i$, and *unidentifiable* if there are infinitely many possible solutions for $p_i$.

Clearly, in this example, we have three unknown parameters and only two identifiable combinations, and therefore, it is not possible to identify all parameters from a single output time course. In fact, solving $\varvec{\zeta }({\textbf {p}})=\varvec{\zeta }(\widetilde{{\textbf {p}}})$ we find that none of the parameters are identifiable individually, and so we conclude that only the grouped parameters

14 $$\begin{aligned} k_{a+}R_{tot}\qquad \text {and}\qquad k_{a+}[A]+k_{a-}\end{aligned}$$

are identifiable.

**Fig. 3:**
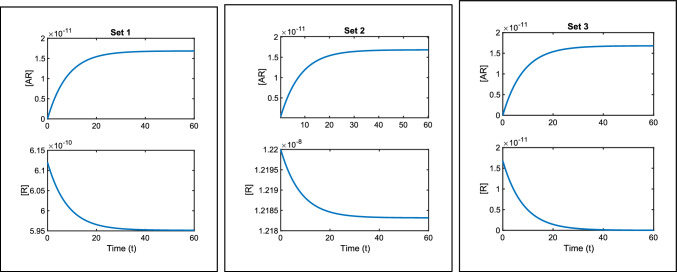
Three sets of parameters are used to plot the system given in Eqs. (3a–d). All three parameter sets give the same measured output curve. However, non-identifiability can be seen in the individual species curves, $[R]$. Each set of plots is created using the values in Table 2 together with $[A]=10^{-8}M$

Non-identifiability for (8a–c) can be seen in the numerical results shown in Fig. 3 that were generated using the parameter values in Table 2. Here we see how, for example, three different parameter sets can result in the same measured output curve ($[AR]$) but have different magnitudes of the free receptor concentrations ($[R]$). In Table 2 we see that the values of the identifiable grouped parameters given in (14) are equal for all three parameter sets.In Sect. 4, we discuss approaches we can take to compensate for the non-identifiability of individual parameters.

**Table 2:** The parameters for the three different parameter sets that are used to plot Fig. 3

	Units	Set 1	Set 2	Set 3
$k_{a+}$	$M^{-1}s^{-1}$	$3.4\times 10^5$	$1.7\times 10^4$	$1.23\times 10^7$
$k_{a-}$	$s^{-1}$	$1.2\times 10^{-1}$	$1.23\times 10^{-1}$	$10^{-5}$
$R_{tot}$	*M*	$6.12\times 10^{-10}$	$1.22\times 10^{-8}$	$1.68\times 10^{-11}$
$k_{a+}R_{tot}$	$s^{-1}$	$2.07\times 10^{-4}$	$2.07\times 10^{-4}$	$2.07\times 10^{-4}$
$k_{a+}[A]+k_{a-}$	$s^{-1}$	$1.23\times 10^{-1}$	$1.23\times 10^{-1}$	$1.23\times 10^{-1}$

The identifiable parameter combination expressions in (14) are equal in each case

### Taylor series method

The next method we present makes use of the Taylor series. The Taylor series method was first developed by Pohjanpalo [45], and can be applied to either linear or nonlinear systems. While the receptor theory models we present in this paper are linear, we will later analyse the identifiability properties of a nonlinear system for ligand binding [57]. We note however, that while the Taylor series may be used to determine identifiability of nonlinear systems, the algebra involved in applying the method to these problems can be difficult [10].

To apply this method to our example model, we can use the system in the form given in (3a–d), or the reduced form (as in (8a–c)), with both giving the same results. We choose to use the full system (3a–d). The Taylor series approach exploits the fact that there is a unique Taylor expansion for a given output ${\textbf {y}}(t)$ about $t=t_0$, and so the Taylor coefficients (we refer to these simply as coefficients throughout) give identifiable parameter combinations [13]. It can also be shown that, for linear problems, the maximum number of coefficients needed to determine identifiability is defined as

15 $$\begin{aligned} k_{max}=2n-1, \end{aligned}$$

where *n* is the number of variables [13]. Coefficients beyond the first $(2n-1)$ terms in the Taylor series give no further information about identifiability.

In our example of monomer-ligand binding (3a–d) we have $n=2$, and will therefore need to calculate a maximum of three coefficients. Here, our initial conditions are taken at time $t=0$, hence we take $t_{0}=0$. Recall, the Taylor series of ${\textbf {y}}$ about $t=0$, noting that we use the bracketed superscript to indicate the order of the derivative, is

16 $$\begin{aligned} {\textbf {y}}(t)={\textbf {y}}(0)+t\,{\textbf {y}}^{(1)}(0)+\frac{t^2}{2!}\,{\textbf {y}}^{(2)}(0)+ \cdots . \end{aligned}$$

The first coefficient is simply

17 $$\begin{aligned} {\textbf {y}}(0). \end{aligned}$$

As $y=[AR]$, we use the initial conditions to obtain the first coefficient as

18 $$\begin{aligned} {\textbf {y}}(0)=[AR](0)=0. \end{aligned}$$

Since the expression for ${\textbf {y}}(0)$ contains no parameters, it gives no information about parameter identifiability. Since we have two remaining coefficients to determine but three unknown parameters, we can immediately conclude that the system is not globally identifiable. However, we will still continue to determine which, if any, parameters are identifiable and also any identifiable parameter combinations. The next coefficient, ${\textbf {y}}^{(1)}(0)$, involves calculating the first derivative of the output function. Now,

19 $$\begin{aligned} {\textbf {y}}^{(1)}(t)=[AR]^{(1)}(t). \end{aligned}$$

From (3b), we have

20 $$\begin{aligned} {\textbf {y}}^{(1)}=k_{a+}[A][R]-k_{a-}[AR]. \end{aligned}$$

Evaluating at $t=0$, using the initial conditions, then gives the second coefficient as

21 $$\begin{aligned} {\textbf {y}}^{(1)}(0)=k_{a+}[A]R_{tot}. \end{aligned}$$

Since $[A]$ is known, we have

22 $$\begin{aligned} c_1=k_{a+}R_{tot}, \end{aligned}$$

where we denote $c_i$ as the $i^{\text {th}}$ identifiable parameter combination. Further derivatives are found with recursive substitution of the equations in system (3a–d). This gives the second derivative as

23 $$\begin{aligned} {\textbf {y}}^{(2)}=[AR]^{(2)}&=k_{a+}[A][R]^{(1)}-k_{a-}[AR]^{(1)} \nonumber \\&=-k_{a+}[A](k_{a+}[A][R]-k_{a-}[AR])-k_{a-}(k_{a+}[A][R]-k_{a-}[AR]) \nonumber \\&=-k_{a+}[A](k_{a+}[A]+k_{a-})[R]+k_{a-}(k_{a+}[A]+k_{a-})[AR]. \end{aligned}$$

After evaluation at $t=0$ and substitution of the initial conditions (3c), the second coefficient is found to be

24 $$\begin{aligned} {\textbf {y}}^{(2)}(0)=-k_{a+}[A]R_{tot}(k_{a+}[A]+k_{a-}). \end{aligned}$$

Using (22), we see that

25 $$\begin{aligned} {\textbf {y}}^{(2)}(0)=-c_1[A](k_{a+}[A]+k_{a-}), \end{aligned}$$

and since $[A]$ is known, we may write

26 $$\begin{aligned} c_2=k_{a+}[A]+k_{a-}, \end{aligned}$$

as the second identifiable parameter combination. Hence, we have two identifiable parameter combinations. Moreover, these agree with the parameter combinations found when using the transfer function method, which is as we expected. To determine which parameters (if any) are identifiable, we would proceed as in the transfer function method by creating the vector $\varvec{\zeta }({\textbf {p}})=(c_1,c_2)$ and solving $\varvec{\zeta }({\textbf {p}})=\varvec{\zeta }(\widetilde{{\textbf {p}}})$.

Calculating further derivatives gives no further identifiable parameter combinations. For example, the third derivative is, after some simplification, given by

27 $$\begin{aligned} y^{(3)}=k_{a+}[A](k_{a+}[A]+k_{a-})^2[R]+k_{a-}(k_{a+}[A]+k_{a-})^2[AR]. \end{aligned}$$

This gives the next Taylor coefficient as

28 $$\begin{aligned} y^{(3)}(0)=k_{a+}[A]R_{tot}(k_{a+}[A]+k_{a-})^2=c_1[A]c_2^2, \end{aligned}$$

and so is comprised of already identifiable parameters and parameter combinations.

### Similarity transformation method

The similarity transformation method (or exhaustive modelling approach) was first proposed by Walter and Lecourtier [53], and originally was only applicable to linear problems. This was later extended to include nonlinear problems [50]. The theory behind this method, along with proofs, can be found in [50].

In order to use the similarity transformation method, the system must be both controllable and observable [33]. A system is said to be controllable if changing the input to the system changes the system states ${\textbf {x}}$, and observable if the initial state ${\textbf {x}}_0$ can be uniquely determined from a set of input–output measurements. In [50], a test for linear systems to determine whether the system is controllable and observable is outlined. For a linear system of the form (9), the controllability matrix $\mathcal {C}$ is defined by

29 $$\begin{aligned} \mathcal {C}=\left[ G \, \vdots \, FG \, \vdots \, F^2G \, \vdots \, \cdots \, \vdots \, F^{n-1}G\right] , \end{aligned}$$

and the observability matrix by

30 $$\begin{aligned} \mathcal {O}=\begin{bmatrix} H \\ HF \\ HF^2 \\ \vdots \\ HF^{n-1} \end{bmatrix}. \end{aligned}$$

The system is then said to be controllable if $\text {rank}(\mathcal {C})=n$, and observable if $\text {rank}(\mathcal {O})=n$.

For our ligand binding model, we consider the system given by (9, 10), where the system input is captured in the ODE. That is, we have

31 $$\begin{aligned} F=-(k_{a+}[A]+k_{a-}),\quad G=k_{a+}[A]R_{tot},\quad H=1, \end{aligned}$$

and $n=1$. As $n=1$, the system is both controllable and observable, hence we now move forward with checking identifiability.

The similarity transform method involves taking this system $\sum ({\textbf {p}})$, and assuming the existence of a system $\sum (\widetilde{{\textbf {p}}})$ that depends on an alternate parameter set $\widetilde{{\textbf {p}}}$. These systems must be *equivalent* (that is, they give the same solution), and so must satisfy some equivalence conditions. The underlying theory and full algebraic equivalence theory can be found in [20, 47] here we just state the conditions. We assume that there exists an $n\times n$ matrix, *T*, that describes the transformation between the two systems. The conditions we impose are then

32a $$\begin{aligned}&\det T \quad \ne 0, \end{aligned}$$

32b $$\begin{aligned}&T\widetilde{{\textbf {x}}}_0 = {\textbf {x}}_0, \end{aligned}$$

32c $$\begin{aligned}&T\widetilde{F}=FT, \end{aligned}$$

32d $$\begin{aligned}&T\widetilde{G}=G, \end{aligned}$$

32e $$\begin{aligned}&\widetilde{H}=HT, \end{aligned}$$

where a tilde indicates the alternate system. Applying these constraints on the two systems determines the identifiability of parameters.

In our system, we have $n=1,$ and so *T* is a single entry matrix. As $H=1$, applying condition (32e) gives

33 $$\begin{aligned} \widetilde{H}=HT,\quad \Rightarrow \quad 1=1\cdot T,\quad \Rightarrow \quad T=1. \end{aligned}$$

Clearly, $\det T\ne 0$ and, as ${\textbf {x}}_0$ is independent of parameters, $T\widetilde{{\textbf {x}}}_0 = {\textbf {x}}_0$, and so conditions (32a) and (32b) both hold. Applying condition (32d), we have

34 $$\begin{aligned} T\widetilde{G}=G,\quad \Rightarrow \quad \widetilde{k_{a+}}[A]\widetilde{R_{tot}}=k_{a+}[A]R_{tot},\quad \Rightarrow \quad \widetilde{k_{a+}}\widetilde{R_{tot}}=k_{a+}R_{tot}. \end{aligned}$$

Finally, we have, from condition (32c)

35 $$\begin{aligned} T\widetilde{F}=FT,&\Rightarrow \quad -(\widetilde{k_{a+}}[A]+\widetilde{k_{a-}})=-(k_{a+}[A]+k_{a-}), \nonumber \\&\Rightarrow \quad \widetilde{k_{a+}}[A]+\widetilde{k_{a-}}=k_{a+}[A]+k_{a-}. \end{aligned}$$

Hence, we find the same identifiable parameter combinations as in the previous methods, with no individually identifiable parameters, confirming that all methods give the same results.

## Results: SIA for further ligand binding models

Now that we have outlined the methods, we will apply each of these methods to the classical Motulsky-Mahan [44] competition binding model, in Sect. 3.1, and to our recently presented dimeric receptor binding model in Sect. 3.2. This will provide a tutorial of how each of the methods work in practice and also allows us to compare the different techniques.

### Competition binding model

The Motulsky-Mahan [44] model is for a competitive binding scenario where two ligands, *A* and *B*, are each able to bind to a monomeric receptor. The ligand concentrations are assumed constant, and each ligand is able to bind the same receptor. Ligand *A* is radiolabelled and the total concentration of *A* bound to receptor can be measured experimentally. Ligand *B* is an unlabelled competitor whose total receptor-bound concentration cannot be measured directly. The reactions describing the interactions are:

$$\begin{array}{*{20}l} {{\text{A}}{\mkern 1mu} + {\mkern 1mu} {\text{R}}{\mkern 1mu} \mathop {\mathop \rightleftharpoons \limits^{{k_{{a + }} }} }\limits_{{k_{{a - }} }} {\mkern 1mu} {\text{AR}},} & {{\text{B}}{\mkern 1mu} + {\mkern 1mu} {\text{R}}{\mkern 1mu} \mathop {\mathop \rightleftharpoons \limits^{{k_{{b + }} }} }\limits_{{k_{{b - }} }} {\mkern 1mu} {\text{BR}}} \\ \end{array}.$$

The system of ODEs governing the dynamics of the system is

36a $$\begin{aligned}{}[R]'&= -(k_{a+}[A]+k_{b+}[B])[R]+k_{a-}[AR]+k_{b-}[BR], \end{aligned}$$

36b $$\left[ {AR} \right]^{\prime } \, = \,k_{{a + }} \left[ A \right]\left[ R \right] - \,k_{{a - }} \left[ {AR} \right],$$

36c $$\left[ {BR} \right]^{\prime } \, = \,k_{{b + }} \left[ A \right]\left[ R \right] - \,k_{{b - }} \left[ {BR} \right],$$

together with the initial conditions

36d $$\begin{aligned}{}[R](0)=R_{tot},\qquad [AR](0)=0, \qquad [BR](0)=0, \end{aligned}$$

where $R_{tot}$ is the total receptor concentration. This forms the initial value problem describing the kinetics of the system. We assume that only the concentration of $[AR]$ is measured experimentally, hence the output is

36e $$\begin{aligned} y=[AR]. \end{aligned}$$

The ligand concentrations $[A]$ and $[B]$ are known constants, and so a vector of unknown parameters in the model is ${\textbf {p}}=(k_{a+},k_{a-},k_{b+},k_{b-},R_{tot})$. In Sect. 4.2, we will consider the scenario suggested in [44] where $k_{a+}$ and $k_{a-}$ are already known from other experiments, but here we analyse the competition binding model with respect to the identifiability of all five parameters using a single timecourse.

#### Transfer function method

We first apply the transfer function method to determine identifiability of the parameters. In keeping with the Motulsky-Mahan analysis [44], we reduce the system in (36a–e) using the conservation law $R_{tot}=[R]+[AR]+[BR]$. This gives

37a $$\left[ {AR} \right]^{\prime } \, = \, - \left( {k_{a} + \left[ A \right]{\text{ }} + {\text{ }}k_{a} - } \right)\left[ {AR} \right]{\text{ }} - {\text{ }}k_{a} + \left[ A \right]\left[ {BR} \right]{\text{ }} + {\text{ }}k_{a} + \left[ A \right]R_{{tot}} ,$$

37b $$\left[ {BR} \right]^{\prime } \, = \, - k_{{b + }} \left[ B \right]\left[ {AR} \right] - \left( {k_{{b + }} \left[ B \right] + k_{{b - }} } \right)\left[ {BR} \right]\, + \,k_{{b + }} \left[ B \right]R_{{tot}} .$$

To write in the form (9), i.e.,

$$\begin{aligned} \sum =\begin{bmatrix} {\textbf {x}}'=F{\textbf {x}}+G{\textbf {u}} \\ {\textbf {y}}=H{\textbf {x}} \\ {\textbf {x}}(0)={\textbf {x}}_0 \end{bmatrix}, \end{aligned}$$

we introduce the matrices

38 $$\begin{aligned} F=\begin{bmatrix} -(k_{a+}[A]+k_{a-}) &{} -k_{a+}[A]\\ -k_{b+}[B]&{} -(k_{b+}[B]+k_{b-}) \end{bmatrix},\quad G=\begin{bmatrix} k_{a+}[A]R_{tot}\\ k_{b+}[B]R_{tot}\end{bmatrix},\quad H=[1,0]. \end{aligned}$$

To check the identifiability of the system, we calculate the transfer function as

39 $$\begin{aligned}&Q(s,{\textbf {p}})= \nonumber &\frac{k_{a+}[A]R_{tot}s+k_{a+}k_{b-}[A]R_{tot}}{s^2+(k_{a+}[A]+k_{b+}[B]+k_{a-}+k_{b-})s+k_{a+}k_{b-}[A]+k_{a-}k_{b+}[B]+k_{a-}k_{b-}}, \end{aligned}$$

which gives the vector of coefficients as

40 $$\begin{aligned} \varvec{\zeta }({\textbf {p}})=\begin{bmatrix} k_{a+}[A]R_{tot}\\ k_{a+}k_{b-}[A]R_{tot}\\ k_{a+}[A]+k_{b+}[B]+k_{a-}+k_{b-}\\ k_{a+}k_{b-}[A]+k_{a-}k_{b+}[B]+k_{a-}k_{b-}\end{bmatrix}. \end{aligned}$$

We have five unknown parameters and only four identifiable combinations, therefore we can immediately conclude that it is not possible for all five parameters to be globally identifiable. Setting $\varvec{\zeta }({\textbf {p}})=\varvec{\zeta }(\widetilde{{\textbf {p}}})$ allows us to determine identifiability of any individual parameters. The first two equations are

41 $$\begin{aligned} k_{a+}[A]R_{tot}&= \widetilde{k_{a+}}[A]\widetilde{R_{tot}}, \end{aligned}$$

42 $$\begin{aligned} k_{a+}k_{b-}[A]R_{tot}&= \widetilde{k_{a+}}\widetilde{k_{b-}}[A]\widetilde{R_{tot}}. \end{aligned}$$

Dividing (42) by (41) gives

43 $$\begin{aligned} k_{b-}= \widetilde{k_{b-}}. \end{aligned}$$

Hence, we find $k_{b-}$ to be identifiable, however, this is the only parameter to be so. This leaves the remaining identifiable parameter combinations as

44 $$\begin{aligned} k_{a+}R_{tot}, \qquad k_{a+}[A]+k_{b+}[B]+k_{a-}, \qquad k_{a+}k_{b-}[A]+k_{a-}k_{b+}[B]+k_{a-}k_{b-}. \end{aligned}$$

**Fig. 4:**
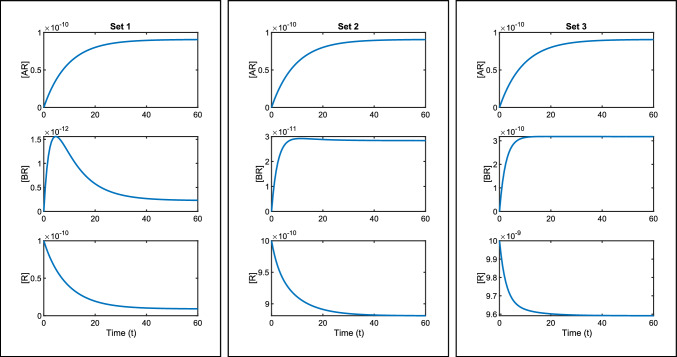
Three sets of parameters are used to plot the solutions of the system (36a–e). All three parameter sets give the same measured output curve, *AR*. However, non-identifiability can be seen in the individual species curves. Each set of plots is created using the values in Table 3 together with $[A]=10^{-8}$M and $[B]=10^{-7}$M

In Fig. 4 the non-identifiability of the model is clear, as three parameter sets (as given in Table 3) result in different species timecourse curves, yet the measured output curve of $[AR]$ is the same for all sets. In particular, we notice that $[BR](t)$ has significant peaks in some of the curves, depending on the parameters used.

**Table 3:** The values of three different parameter sets that are used to plot Fig. 4

Parameter	Units	Set 1	Set 2	Set 3
$k_{a+}$	M^-1^s^-1^	$10^7$	$10^6$	$10^5$
$k_{a-}$	s^-1^	0.01	0.097123	0.105746
$k_{b+}$	M^-1^s^-1^	$10^{5}$	128765.3	132538.6
$k_{b-}$	s^-1^	0.4	0.4	0.4
$R_{tot}$	M	$10^{-10}$	$10^{-9}$	$10^{-8}$
$k_{a+}R_{tot}$	s^-1^	$10^{-3}$	$10^{-3}$	$10^{-3}$
$k_{a+}[A]+k_{b+}[B]+k_{a-}$	s^-1^	0.12	0.12	0.12
$k_{a+}k_{b-}[A]+k_{a-}k_{b+}[B]+k_{a-}k_{b-}$	s^-2^	0.0441	0.0441	0.0441

The parameter $k_{b-}$, as well as the parameter combinations in the final three rows are equal in each case, as these are the identifiable quantities

#### Taylor series method

To apply the Taylor series we consider the system (36a–e). As we now have three state variables, we have $n=3$, so (15) gives that there is a maximum of five coefficients required to determine identifiability. Calculating the Taylor series coefficients, as stated in (16), and evaluating at $t = 0$, gives the identifiable parameter combinations. Again, the first coefficient is

45 $$\begin{aligned} y_0=0, \end{aligned}$$

which gives no information regarding identifiability. As we have five unknown parameters and only four remaining coefficients to evaluate, we can conclude that the system is not globally identifiable. The first derivative, as given by (36b), is

46 $$\begin{aligned} y^{(1)}(t)=k_{a+}[A][R]-k_{a-}[AR], \end{aligned}$$

giving the first Taylor coefficient as

47 $$\begin{aligned} y^{(1)}(0)=k_{a+}[A]R_{tot}, \end{aligned}$$

and as such, the first identifiable parameter combination as

48 $$\begin{aligned} c_1=k_{a+}R_{tot}, \end{aligned}$$

since $[A]$ is known. Using recursive substitution of equations (36a–e), we can write the second derivative as

49 $$\begin{aligned} y^{(2)}&=-k_{a+}[A]((k_{a+}[A]+k_{b+}[B])[R]-k_{a-}[AR]-k_{b-}[BR]) \nonumber \\&\quad -k_{a-}(k_{a+}[A][R]-k_{a-}[AR]) \nonumber \\&=-k_{a+}[A](k_{a+}[A]+k_{b+}[B]+k_{a-})[R]\nonumber \\&\quad + k_{a-}(k_{a-}+k_{a+}[A])[AR]+k_{a+}k_{b-}[A][BR], \end{aligned}$$

which gives the coefficient

50 $$\begin{aligned} y^{(2)}(0)=k_{a+}[A]R_{tot}(k_{a+}[A]+k_{b+}[B]+k_{a-}). \end{aligned}$$

Now,

51 $$\begin{aligned} y^{(2)}(0)&=k_{a+}[A]R_{tot}(k_{a+}[A]+k_{b+}[B]+k_{a-}), \end{aligned}$$

52 $$\begin{aligned} y^{(2)}(0)&=c_1[A](k_{a+}[A]+k_{b+}[B]+k_{a-}), \end{aligned}$$

and therefore, we have a second identifiable combination as

53 $$\begin{aligned} c_2=k_{a+}[A]+k_{b+}[B]+k_{a-}. \end{aligned}$$

Further coefficients are found in the same way, using recursive substitution of the system equations in (36a–e) to calculate higher order derivatives followed by substitution of the initial conditions. Using this method we obtain the third coefficient as

54 $$\begin{aligned} y^{(3)}(0)&=k_{a+}[A]R_{tot}(k_{a+}^2[A]^2+2k_{a+}k_{b+}[A][B]+2k_{a+}k_{a-}[A]\nonumber \\&\quad +k_{b+}^2[B]^2+k_{a-}k_{b+}[B]+k_{b+}k_{b-}[B]+k_{a-}^2) \nonumber \\&=c_1[A](c_2^2+k_{b+}(k_{a-}-k_{b-})), \end{aligned}$$

which gives the third identifiable combination as

55 $$\begin{aligned} c_3=k_{b+}(k_{a-}-k_{b-}). \end{aligned}$$

We also obtain

56 $$\begin{aligned} y^{(4)}(0)&=k_{a+}[A]R_{tot}(k_{a+}^4[A]^4+4k_{a+}^3k_{b+}[A]^3[B]+4k_{a+}^3k_{a-}[A]^3 \nonumber \\&\quad + 6k_{a+}^2k_{b+}^2[A]^2[B]^2 \nonumber \\&\quad +9k_{a+}^2k_{a-}k_{b+}[A]^2[B]+3k_{a+}^2k_{b+}k_{b-}[A]^2[B]\nonumber \\&\quad +6k_{a+}^2k_{a-}^2[A]^2+4k_{a+}k_{b+}^3[A][B]^3 \nonumber \\&\quad +6k_{a+}k_{a-}k_{b+}^2[A][B]^2 +6k_{a+}k_{b+}^2k_{b-}[A][B]^2 \nonumber \\&\quad +6k_{a+}k_{a-}^2k_{b+}[A][B]+4k_{a+}k_{a-}k_{b+}k_{b-}[A][B]\nonumber \\&\quad +2k_{a+}k_{b+}k_{b-}^2[A][B]+4k_{a+}k_{a-}^3[A]+k_{b+}^4[B]^4 \nonumber \\&\quad +k_{a-}k_{b+}^3[B]^3+3k_{b+}^3k_{b-}[B]^3+k_{a-}^2k_{b+}^2[B]^2 \nonumber \\&\quad +2k_{a-}k_{b+}^2k_{b-}[B]^2+3k_{b+}^2k_{b-}^2[B]^2+k_{a-}^3k_{b+}[B]\nonumber \\&\quad +k_{a-}^2k_{b+}k_{b-}[B]+k_{a-}k_{b+}k_{b-}^2[B]\nonumber \\&\quad +k_{b+}k_{b-}^2[B]+k_{b+}k_{b-}^3[B]+k_{a-}^4), \end{aligned}$$

which, we find via some trial and error, may be written as

57 $$\begin{aligned} y^{(4)}(0)=c_1(c_2^3+c_3(2c_2+k_{b-})), \end{aligned}$$

which gives the identifiable parameter

58 $$\begin{aligned} c_4=k_{b-}. \end{aligned}$$

We note that the calculations are all performed using MATLAB Symbolic Toolbox [40]. To conclude, we find that $k_{b-}$ is identifiable, and also the identifiable combinations

59 $$\begin{aligned} \varvec{\zeta }({\textbf {p}})=\begin{bmatrix} k_{a+}R_{tot}\\ k_{b+}(k_{a-}-k_{b-}) \\ k_{a+}[A]+k_{b+}[B]+k_{a-}\end{bmatrix}. \end{aligned}$$

Although these parameter combinations are not identical to those found from the transfer function method in expression (44), we note that

60 $$\begin{aligned} k_{b+}(k_{a-}-k_{b-})+k_{b-}(k_{a+}[A]+k_{b+}[B]+k_{a-})=k_{a+}k_{b-}[A]+k_{a-}k_{b+}[B]+k_{a-}k_{b-}, \end{aligned}$$

hence the same four parameter combinations are indeed identifiable.

#### Similarity transformation method for competition binding model

In Appendix 1, we apply the similarity transform method, as introduced in Sect. 2.3, to the Motulsky-Mahan competition binding system. We show that the same parameter combinations are found to be identifiable as for the previous methods (see (44) and (59)).

Comparing the methods, it is clear that, although all methods give the same identifiable parameters and parameter combinations, the transfer function method is by far the simplest in terms of ease of use. The Taylor series method, in particular, results in expressions that require quite some manipulation in order to obtain reduced expressions.

### Pre-dimerised G protein-coupled receptor binding

The next model, and the final linear model, we consider is the GPCR homodimer model we presented and analysed in [55], for a single ligand binding. The schematic for the model is as follows.

$$\begin{array}{*{20}c} {{\text{A}}{\mkern 1mu} + {\mkern 1mu} {\text{R}}\,{\mkern 1mu} \mathop {\mathop \rightleftharpoons \limits^{{k_{{a + }} }} }\limits_{{k_{{a - }} }} {\mkern 1mu} \,{\text{AR}},\,} & {{\text{A}}{\mkern 1mu} + {\mkern 1mu} {\text{AR}}\,{\mkern 1mu} \mathop {\mathop \rightleftharpoons \limits^{{\alpha _{ + } k_{{a + }} }} }\limits_{{\alpha _{ - } k_{{a - }} }} {\mkern 1mu} \,{\text{ARA}}{\text{.}}} \\ \end{array}$$

Here, *R* represents the dimerised receptor, *AR* is the dimerised receptor with one ligand bound, and *ARA* is the dimerised receptor with both protomers bound by ligand. The parameters $\alpha _{+}$ and $\alpha _{-}$ are the forwards and backwards binding cooperativities respectively. These capture the increased or decreased propensity for binding and dissociation when the opposite side of the dimer is ligand-bound rather than unoccupied.

The ODE system describing the model dynamics is given by (see [55])

61a $$\begin{aligned} \frac{d[R]}{dt}&=-k_{a+}[A][R]+k_{a-}[AR], \end{aligned}$$

61b $$\begin{aligned} \frac{d[AR]}{dt}&= k_{a+}[A][R]-(k_{a-}+\alpha _+k_{a+}[A])[AR]+\alpha _-k_{a-}[ARA], \end{aligned}$$

61c $$\begin{aligned} \frac{d[ARA]}{dt}&=\alpha _+k_{a+}[A][AR]-\alpha _-k_{a-}[ARA], \end{aligned}$$

with initial conditions

61d $$\begin{aligned}{}[R](0)=R_{tot},\qquad [AR](0)=0,\qquad [ARA](0)=0. \end{aligned}$$

The measured quantity is bound ligand, hence the output is given by

61e $$\begin{aligned} y=[AR]+2[ARA]. \end{aligned}$$

We assume the only known parameter is the ligand concentration, $[A]$, and so we have the vector of unknown parameters as ${\textbf {p}}=(\alpha _+,\alpha _-,k_{a+},k_{a-},R_{tot})$, where $R_{tot}$ is total dimerised receptor. In contrast to the monomeric receptor output (3d), we note that the output function is now a combination of two states, adding a significant difference to the proceeding computations.

#### Transfer function method

Again, we consider the transfer function method to determine identifiability. We first use conservation of receptors, which is given in this case by

62 $$\begin{aligned} R_{tot}=[R]+[AR]+[ARA], \end{aligned}$$

to reduce the system, giving

63a $$\begin{aligned} \frac{d[AR]}{dt}&=-(k_{a+}[A]+k_{a-}+\alpha _+k_{a+}[A])[AR]\nonumber \\&\quad + (\alpha _-k_{a-}-k_{a+}[A])[ARA]+ k_{a+}[A]R_{tot}, \end{aligned}$$

63b $$\begin{aligned} \frac{d[ARA]}{dt}&= \alpha _+k_{a+}[A][AR]- \alpha _-k_{a-}[ARA]. \end{aligned}$$

This is in the form (9), where we identify

64 $$\begin{aligned} F&=\begin{bmatrix} -(k_{a+}[A]+k_{a-}+\alpha _+k_{a+}[A]) &{} \alpha _-k_{a-}-k_{a+}[A]\\ \alpha _+k_{a+}[A]&{} -\alpha _-k_{a-}\end{bmatrix}, \nonumber \\&\qquad G=\begin{bmatrix} k_{a+}[A]R_{tot}\\ 0 \end{bmatrix},\qquad H=\begin{bmatrix} 1&2 \end{bmatrix}. \end{aligned}$$

We find the transfer function of the system to be

65 $$\begin{aligned}&Q(s,{\textbf {p}})= \nonumber &\frac{k_{a+}[A]R_{tot}(s+2\alpha _+k_{a+}[A]+\alpha _-k_{a-})}{s^2+(k_{a+}[A]+k_{a-}+\alpha _+k_{a+}[A]+\alpha _-k_{a-})s+(\alpha _+k_{a+}^2[A]^2+\alpha _-k_{a+}k_{a-}[A]+\alpha _-k_{a-}^2)}, \end{aligned}$$

which gives the vector of identifiable parameter combinations as

66 $$\begin{aligned} \varvec{\zeta }({\textbf {p}})=\begin{bmatrix} k_{a+}R_{tot}\\ 2\alpha _+k_{a+}[A]+\alpha _-k_{a-}\\ k_{a+}[A]+k_{a-}+\alpha _+k_{a+}[A]+\alpha _-k_{a-}\\ \alpha _+k_{a+}^2[A]^2+\alpha _-k_{a+}k_{a-}[A]+\alpha _-k_{a-}^2 \end{bmatrix}. \end{aligned}$$

**Fig. 5:**
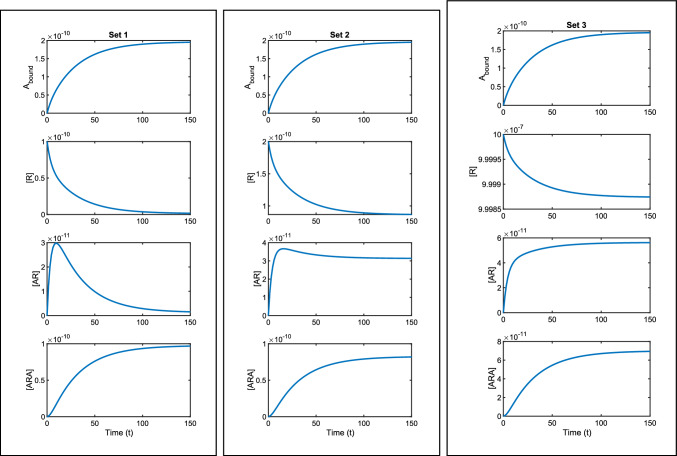
Three sets of parameters are used to plot the solution to equations (61a–e). All three parameter sets give the same measured output curve, $A_{bound}$. However, non-identifiability can be seen in the individual species curves. Each set of plots is created using the values in Table 4 together with $[A]=10^{-8}$M

Hence we have no identifiable parameters but do have four identifiable parameter combinations. Again this can be seen in Fig. 5 where we show how three sets of different parameter values result in different individual species curves, yet all give the same measured output curve $A_{bound}$. While the curves of $[R]$ and $[ARA]$ are similar in shape across the three parameter sets, they have different magnitudes of concentration. The clearest differences are seen in the $[AR]$ curves, where the different parameter sets result in curves that have distinctly different evolution patterns, with some curves having a peak and fall while others are monotonic. This highlights how naive parameter estimation performed without knowledge of identifiability issues could lead to incorrect conclusions being drawn about the underlying qualitative dynamics. The parameter values used for the plots are given in Table 4.

**Table 4:** The parameters for three different parameter sets are used to plot Fig. 5

Parameter	Units	Set 1	Set 2	Set 3
$k_{a+}$	M^-1^s^-1^	$10^7$	$5\times 10^6$	$10^3$
$k_{a-}$	s^-1^	0.1	0.137667	0.177379
$\alpha _+$	–	0.8	1.353341	5738.851
$\alpha _-$	–	0.01	0.186435	0.260590
$R_{tot}$	M	$10^{-10}$	$2\times 10^{-10}$	$10^{-6}$
$k_{a+}R_{tot}$	s^-1^	$10^{-3}$	$10^{-3}$	$10^{-3}$
$2\alpha _+k_{a+}[A]+\alpha _-k_{a-}$	s^-1^	0.161	0.161	0.161
$k_{a+}[A]+k_{a-}+\alpha _+k_{a+}[A]+\alpha _-k_{a-}$	s^-1^	0.281	0.281	0.281
$\alpha _+k_{a+}^2[A]^2+\alpha _-k_{a+}k_{a-}[A]+\alpha _-k_{a-}^2$	s^-2^	0.0082	0.0082	0.0082

The parameter combinations in the final four rows are equal in each case; these are the identifiable combinations found in (66)

#### Taylor series method

We proceed with the Taylor series method to determine identifiability, with repeated substitution of the ODEs and initial conditions. While the process is the same as for the competition binding model in Sect. 3.1, the output function being a combination of two state variables adds an extra complexity to the calculations. As in all previous sections, the first coefficient is trivial, that is

67 $$\begin{aligned} y_0=0. \end{aligned}$$

The first derivative of the output function in (61e) is given by

68 $$\begin{aligned} y^{(1)}&=k_{a+}[A][R]-(k_{a-}+\alpha _+k_{a+}[A])[AR]+\alpha _-k_{a-}[ARA]\nonumber \\&\qquad +2(\alpha _+k_{a+}[A][AR]-\alpha _-k_{a-}[ARA]) \nonumber \\&=k_{a+}[A][R]-(k_{a-}-\alpha _+k_{a+}[A])[AR]-\alpha _-k_{a-}[ARA], \end{aligned}$$

which, using the initial conditions in (61d), gives the first unique coefficient as

69 $$\begin{aligned} y^{(1)}(0)=k_{a+}[A]R_{tot}. \end{aligned}$$

The remaining three coefficients are calculated by following the method of Sect. 3.1.2, by repeatedly differentiating the output expression and substituting in the dynamic equations  (61a–e). We find that

70 $$\begin{aligned} y^{(2)}(0)&=k_{a+}[A]R_{tot}(\alpha _+k_{a+}[A]-k_{a+}[A]-k_{a-}), \end{aligned}$$

71 $$\begin{aligned} y^{(3)}(0)&=k_{a+}[A]R_{tot}(k_{a+}^2[A]^2-\alpha _+^2k_{a+}^2[A]^2 \nonumber \\&\qquad -\alpha _+k_{a+}^2[A]^2+2k_{a+}k_{a-}[A]-\alpha _-k_{a+}k_{a-}[A]+k_{a-}^2), \end{aligned}$$

72 $$\begin{aligned} y^{(4)}(0)&=k_{a+}[A]R_{tot}(\alpha _+^3k_{a+}^3[A]^2+\alpha _+^2k_{a+}^3[A]^3+\alpha _+k_{a+}^3[A]^3-k_{a+}^3[A]^3 \nonumber \\&\qquad +2\alpha _+^2\alpha _-k_{a+}^2k_{a-}[A]^2+\alpha _+\alpha _-k_{a+}^2k_{a-}[A]^2 \nonumber \\&\qquad +\alpha _+^2k_{a+}^2k_{a-}[A]^2-3k_{a+}^2k_{a-}[A]^2 \nonumber \\&\qquad +\alpha _+\alpha _-^2k_{a+}k_{a-}^2[A]-\alpha _+k_{a+}k_{a-}^2[A]-3k_{a+}k_{a-}^2[A]-k_{a-}^3). \end{aligned}$$

A vector of identifiable combinations is then given by

73a $$\begin{aligned} \varvec{\zeta }_{1}({\textbf {p}})=\begin{bmatrix} k_{a+}R_{tot}\\ P \\ Q \\ R \end{bmatrix}, \end{aligned}$$

where

73b $$\begin{aligned} P&= \alpha _+k_{a+}[A]-k_{a+}[A]-k_{a-}, \end{aligned}$$

73c $$\begin{aligned} Q&= k_{a+}^2[A]^2-\alpha _+^2k_{a+}^2[A]^2-\alpha _+k_{a+}^2[A]^2+2k_{a+}k_{a-}[A]-\alpha _-k_{a+}k_{a-}[A]+k_{a-}^2, \end{aligned}$$

73d $$\begin{aligned} R&= \alpha _+^3k_{a+}^3[A]^2+\alpha _+^2k_{a+}^3[A]^3+\alpha _+k_{a+}^3[A]^3-k_{a+}^3[A]^3 \nonumber \\&\qquad + 2\alpha _+^2\alpha _-k_{a+}^2k_{a-}[A]^2+\alpha _+\alpha _-k_{a+}^2k_{a-}[A]^2 \nonumber \\&\qquad +\alpha _+^2k_{a+}^2k_{a-}[A]^2-3k_{a+}^2k_{a-}[A]^2 \nonumber \\&\qquad +\alpha _+\alpha _-^2k_{a+}k_{a-}^2[A]-\alpha _+k_{a+}k_{a-}^2[A]-3k_{a+}k_{a-}^2[A]-k_{a-}^3. \end{aligned}$$

While it is not immediately apparent that the Taylor Series method gives the same identifiable combinations as the transfer function method, we show in Appendix 2 that we can recover the four combinations in (66) from those in (73a–d) by algebraic manipulation, aided by symbolic computation. The result, again, is that the identifiable combinations are

74 $$\begin{aligned} \varvec{\zeta }({\textbf {p}})=\begin{bmatrix} k_{a+}R_{tot}\\ 2\alpha _+k_{a+}[A]+\alpha _-k_{a-}\\ k_{a+}[A]+k_{a-}+\alpha _+k_{a+}[A]+\alpha _-k_{a-}\\ \alpha _+k_{a+}^2[A]^2+\alpha _-k_{a+}k_{a-}[A]+\alpha _-k_{a-}^2 \end{bmatrix}. \end{aligned}$$

#### Similarity transformation method for GPCR dimer model

In Appendix 3, we apply the similarity transformation method to the GPCR dimer model, and find that he identifiable parameter combinations are the same as those in (66) and (74).

Comparing the three methods applied to this system, we find that the transfer function method is the most straightforward to implement, whereas the Taylor series method results in expressions that require much simplification.

## Results: addressing identifiability issues with equilibrium, washout and multiple time courses

The results thus far have shown none of the models to be globally identifiable from a single set of time course data. In this section we consider alternative ways in which all parameters can be identified. Commonly performed experiments for ligand binding include equilibrium (or saturation) binding assays, in which equilibrium binding levels are measured for a range of ligand concentrations to produce a concentration-response curve. For each ligand concentration, the binding experiments are run until equilibrium is assumed after which the amount of ligand bound is observed. These experiments are often used to estimate equilibrium constants $K_D=1/K_A$ (the equilibrium dissociation constant), where $K_A=k_{a+}/k_{a-}$, and $R_{tot}$ (total receptor concentration), for monomeric receptors [34]. Here, for each model in Sects. 2, 3, we aim to establish identifiability for the corresponding equilibrium model, then use “known” equilibrium parameters together with timecourse data to establish identifiability for those kinetic parameters which were previously unidentifiable.

Washout experiments can also be used to gain further insights into the dissociation kinetics of ligands. In these experiments the free ligand is removed by repeated washing, ensuring that no further ligand associates with the receptors [41]. Such experiments isolate the effect of dissociation and preclude further binding. Here, we consider this type of experimental dataset to establish identifiability of the kinetic dissociation parameters, in conjunction with association (binding) time course data to also determine identifiability of association parameters.

Finally, we also consider multiple binding experiments, whereby each data set is collected from experiments performed using different ligand concentrations. These data sets are then used simultaneously, with the aim of determining the minimum number of data sets required to make the model globally identifiable. In each case, we choose one identifiability method to apply.

### Monomeric receptor binding with a single ligand

Recall the model for a monomeric receptor binding with a single ligand, as given by the schematic

$${\text{A}}\, + \,{\text{R}}\,\underset{{k_{{a - }} }}{\overset{{k_{{a + }} }}{\rightleftharpoons}}\,{\text{AR}}$$

with the system of equations as given in (3a–d). In Sect. 2, we found that there are no identifiable parameters, only the parameter combinations

75 $$\begin{aligned} \varvec{\zeta }({\textbf {p}})=\begin{bmatrix} k_{a+}[A]R_{tot}\\ k_{a+}[A]+k_{a-}\end{bmatrix} \end{aligned}$$

are identifiable. We will use three different approaches to establish global structural identifiability, namely, concentration-response/saturation data together with a single set of time course data, a combination of association and dissociation data, and also multiple time courses. In each case we use an appropriate method from the three that we outlined in Sect. 2. In most cases this is the transfer function method due to its simplicity of implementation, however, dissociation timecourse data are analysed using the Taylor series method.

#### Equilibrium saturation curves

We first establish the identifiability of equilibrium parameters associated with ligand binding, namely $K_A=1/K_D$ (the equilibrium dissociation constant) and $R_{tot}$ (total receptor). Note that, at equilibrium, as $[AR]'=0$ in (3b), we have

76 $$\begin{aligned}{}[AR]=K_A[A][R], \end{aligned}$$

where

77 $$\begin{aligned} K_A=k_{a+}/k_{a-}. \end{aligned}$$

Substituting in $[R]=R_{tot}-[AR]$ and solving for $[AR]$ gives the usual expression (see also [34], for example) for the concentration of ligand bound at equilibrium as

78 $$\begin{aligned} A_{bound}=\frac{K_A[A]R_{tot}}{1+K_A[A]}. \end{aligned}$$

Taking two ligand concentrations, $[A]_1$ and $[A]_2$, and the corresponding output measurements, $[AR]_1$ and $[AR]_2$ gives

79 $$\begin{aligned} \frac{K_A[A]_1R_{tot}}{1+K_A[A]_1} = [AR]_1 \qquad \text {and}\qquad \frac{K_A[A]_2R_{tot}}{1+K_A[A]_2} = [AR]_2. \end{aligned}$$

These equations contain the two unknown parameters $R_{tot}$ and $K_A=k_{a+}/k_{a-}$. Solving for these gives a unique solution

80 $$\begin{aligned} R_{tot}=\frac{[AR]_1 [AR]_2 ([A]_1-[A]_2)}{[A]_1 [AR]_2-[A]_2 [AR]_1} \qquad \text {and}\qquad K_A=\frac{[A]_1 x_2-[A]_2 x_1}{[A]_1[A]_2([AR]_1-[AR]_2)} \end{aligned}$$

and hence these parameters are identifiable from a single dose-response curve. In fact, only two points on the curve are needed, theoretically. Once these are known, we conclude from (77) that only a single parameter, either $k_{a+}$ or $k_{a-}$, remains to be found. This can be obtained from time course data. Using only one of the parameter combinations in (75), we find

81 $$\begin{aligned} k_{a-}K_A[A]R_{tot}=\widetilde{k_{a-}} K_A[A]R_{tot}\qquad \Rightarrow \qquad k_{a-}=\widetilde{k_{a-}}. \end{aligned}$$

Hence, we conclude that, using equilibrium data (a dose-response curve) together with a single set of time course data, it is possible to identify all three model parameters $(k_{a+},k_{a-},R_{tot})$.

#### Washout experiments

We consider using washout experiment data to identify dissociation parameters. In a washout experiment, the ligand is removed from the system (usually once equilibrium has been reached), hence, we set $[A]=0$ in the model given in equations (3a–d). This gives

82a $$[R]^{\prime}= k_{a-}[AR],$$

82b $$[AR]^{\prime}= -k_{a-}[AR].$$

As the concentration of free receptor is unknown at the start point of washout, we write the initial conditions as

82c $$\begin{aligned}{}[R](0)=R_{tot}-[AR]_w,\qquad [AR](0)=[AR]_w, \end{aligned}$$

where the *w* refers to the value when washout begins, at time $t=0$. The output remains unchanged

82d $$\begin{aligned} y=[AR]. \end{aligned}$$

The unknown parameters in this model are $k_{a-}$ and $R_{tot}$. We note that, it is clearly possible to solve the ODE for $[AR]$, specifically giving

83 $$\begin{aligned}{}[AR](t)=[AR](0)e^{-k_{a-}t}, \end{aligned}$$

and use the result to determine identifiability directly. However, we refrain from this here and continue with our SIA methodology applied to the ODE system in order to highlight the general process.

Here, the Taylor series approach is straightforward (as outlined in Sect. 2.2); no reformulation of the ODE system is required and the unknown initial conditions are naturally incorporated into the analysis. Since the number of variables $n=2$, we need to determine a maximum of three Taylor coefficients (see (15)). Calculating the first of these coefficients gives

84 $$\begin{aligned} y=AR, \qquad \Rightarrow \qquad y_0=[AR]_w, \end{aligned}$$

which clearly gives no information about $k_{a-}$, but does provide the value of $[AR]_w$. The second coefficient is

85 $$\begin{aligned} y^{(1)}=AR'=-k_{a-}[AR], \qquad \Rightarrow \qquad y^{(1)}_0=-k_{a-}[AR]_w, \end{aligned}$$

and so we find $k_{a-}$ to be identifiable. The final coefficient is calculated as

86 $$\begin{aligned} c_1=k_{a-}^2 [AR]_w, \end{aligned}$$

which gives no further information, hence, only $k_{a-}$ is identifiable from washout data (as we would expect, given (83)). Since $R_{tot}$ does not appear in  (84)-(86), it is clearly not identifiable from the washout model alone. Combining the newly established identifiability of $k_{a-}$ with the results we obtained from a binding timecourse, namely the parameter combinations in (75), we find that the remaining parameters, $k_{a+}$ and $R_{tot}$, are now identifiable. Hence the system, considering the combination of both experiments, is now globally identifiable, using experimental data for association and washout for a single ligand concentration.

#### Multiple time courses

Next, we consider the case where, instead of one time course, we have multiple sets of time course data, each with a different ligand concentration. Each of these will individually give the identifiable parameters, as stated in equation (75) for their corresponding concentration of $[A]$. That is, the identifiable parameter combinations are given by

87 $$\begin{aligned} \varvec{\zeta }({\textbf {p}})_i=\begin{bmatrix} k_{a+}[A]_iR_{tot}\\ k_{a+}[A]_i+k_{a-}\end{bmatrix}, \end{aligned}$$

for $i=1,2,...$, for the number of time courses being considered. As fitting may be performed on all sets simultaneously, we analyse the corresponding system as a single system. For example, for two time courses we have

88 $$\begin{aligned} \varvec{\zeta }({\textbf {p}})=\begin{bmatrix} k_{a+}[A]_1R_{tot}\\ k_{a+}[A]_1+k_{a-}\\ k_{a+}[A]_2R_{tot}\\ k_{a+}[A]_2+k_{a-}\end{bmatrix}. \end{aligned}$$

Setting $\varvec{\zeta }({\textbf {p}})=\varvec{\zeta }(\widetilde{{\textbf {p}}})$ results in all parameters being successfully identified. This is shown easily by considering the following system:

89a $$\begin{aligned} k_{a+}[A]_1R_{tot}&= \widetilde{k_{a+}}[A]_1\widetilde{R_{tot}}, \end{aligned}$$

89b $$\begin{aligned} k_{a+}[A]_1+k_{a-}&= \widetilde{k_{a+}}[A]_1+\widetilde{k_{a-}}, \end{aligned}$$

89c $$\begin{aligned} k_{a+}[A]_2R_{tot}&= \widetilde{k_{a+}}[A]_2\widetilde{R_{tot}}, \end{aligned}$$

89d $$\begin{aligned} k_{a+}[A]_2+k_{a-}&= \widetilde{k_{a+}}[A]_2+\widetilde{k_{a-}}. \end{aligned}$$

From (89b,d), we see that

$$\begin{aligned} k_{a+}([A]_{1}-[A]_{2}) = \widetilde{k_{a+}}([A]_{1}-[A]_{2}), \qquad \Rightarrow \qquad k_{a+}=\widetilde{k_{a+}}, \end{aligned}$$

and so $k_{a+}$ is identifiable. Then (89a) gives $R_{tot}=\widetilde{R_{tot}}$ and (89b) gives $k_{a-}=\widetilde{k_{a-}}$, so that all three parameters are identifiable. We conclude that the model is fully identifiable from just two time courses.

### Competition binding model

Next, we consider the model for a monomeric receptor binding with two ligands in a competition binding scenario, with a labelled ligand *A* and an unlabelled ligand *B*. This is described by the schematic

$$\begin{array}{*{20}c} {{\text{A}}{\mkern 1mu} \, + {\mkern 1mu} \,{\text{R}}\,{\mkern 1mu} \mathop {\mathop {\, \rightleftharpoons }\limits^{{k_{{a + }} }} }\limits_{{k_{{a - }} }} {\mkern 1mu} \,\,{\text{AR}},\,} & {{\text{B}}\,{\mkern 1mu} + {\mkern 1mu} \,{\text{R}}\,\,{\mkern 1mu} \mathop {\mathop \rightleftharpoons \limits^{{k_{{b + }} }} }\limits_{{k_{{b - }} }} {\mkern 1mu} \,\,{\text{BR,}}} \\ \end{array}$$

and the related system of equations is given in (36a–e). In Sect. 3.1, we performed the identifiability analysis considering the parameters ${\textbf {p}}=(k_{a+},k_{a-},k_{b+},k_{b-},R_{tot})$, and concluded that from a single time course the parameter $k_{b-}$ is uniquely identifiable, as well as the parameter combinations

90 $$\begin{aligned} \varvec{\zeta }({\textbf {p}})=\begin{bmatrix} k_{a+}R_{tot}\\ k_{a+}[A]+k_{b+}[B]+k_{a-}\\ k_{a+}k_{b-}[A]+k_{a-}k_{b+}[B]+k_{a-}k_{b-}\end{bmatrix}. \end{aligned}$$

A simple analysis towards establishing identifiability of all parameters is suggested by the scenario discussed in [44] where $k_{a+}$ and $k_{a-}$ are already known from other experiments. For example, we can consider $[B]=0$, whereby there is no competition, and use the monomeric receptor model of Sects. 2 and  4.1 to ensure identifiability of these parameters. Then we may treat $k_{a+}$ as known in the first row of (90), meaning that $R_{tot}$ is identifiable. Treating $k_{a+}$ and $k_{a-}$ as known in the second row of (90), we see that $k_{b+}$ also becomes identifiable. Hence, with prior knowledge of $k_{a+}$ and $k_{a-}$, the original system is globally identifiable from a single timecourse with $[B]\ne 0$.

Continuing with our detailed tutorial approach, we now consider experiments which may be used to establish identifiability of $k_{a-}$, $k_{a+}$, $k_{b+}$ and $R_{tot}$ without the prior knowledge of the binding parameters for ligand A. Again we consider equilibrium concentration-response, a washout timecourse curve or a second timecourse.

#### Saturation curves

Here, we combine the above results with an equilibrium concentration-response curve. It follows from system (36a–e) that at equilibrium, we have the relations

91 $$\begin{aligned}{}[AR]=K_A[A][R],\qquad [BR]=K_B[B][R], \end{aligned}$$

where $K_A=k_{a+}/k_{a-}$ and $K_B=k_{b+}/k_{b-}$. Moreover, we have the conservation law

92 $$\begin{aligned}{}[R]+[AR]+[BR]=R_{tot}. \end{aligned}$$

Combining these and solving for $[AR]$ gives the equilibrium concentration of the measured ligand bound as

93 $$\begin{aligned}{}[AR]=\frac{K_A[A]R_{tot}}{1+K_A[A]+K_B[B]}. \end{aligned}$$

Assuming we have a concentration-response curve, with a fixed concentration of ligand $[B]$, then we take two points on this curve, giving

94 $$\begin{aligned} \frac{K_A[A]_iR_{tot}}{1+K_A[A]_i+K_B[B]}=[AR]_i, \end{aligned}$$

for $i=1,2$. This results in a system of two equations for $K_A$ and $R_{tot}$, which has the following solution:

95 $$\begin{aligned} K_A&=\frac{(1+K_B[B])([A]_1[AR]_2-[A]_2[AR]_1)}{[A]_1[A]_2([AR]_1-[AR]_2)}, \nonumber \\ R_{tot}&=\frac{[AR]_1[AR]_2([A]_1-[A]_2)}{[A]_1[AR]_2-[A]_2[AR]_1}. \end{aligned}$$

Considering further points on the dose-response curve gives no extra information. Hence from a single concentration-response curve the only identifiable parameter is $R_{tot}$, since the expression for $K_A$ still depends on the unknown parameter $K_B$.

We combine the above with the results from a time course dataset, as given by the parameter combinations in (90). Since $R_{tot}$ and $k_{b-}$ are now known, the unknowns are given by ${\textbf {p}}=(k_{a+},k_{a-},k_{b+})$. We consider $\varvec{\zeta }({\textbf {p}})=\varvec{\zeta }(\widetilde{{\textbf {p}}})$, where $\varvec{\zeta }$ is given in (90). The first equation can be solved as

96 $$\begin{aligned} k_{a+}R_{tot}=\widetilde{k_{a+}}R_{tot},\qquad \Rightarrow \qquad k_{a+}=\widetilde{k_{a+}}, \end{aligned}$$

thus $k_{a+}$ is now identifiable. The other two resultant equations, using that $k_{b-}$ and $k_{a+}$ are known, are

97 $$\begin{aligned} k_{b+}[B]+k_{a-}&= \widetilde{k_{b+}}[B]+\widetilde{k_{a-}}, \end{aligned}$$

98 $$\begin{aligned} k_{a-}k_{b+}[B]+k_{a-}k_{b-}&= \widetilde{k_{a-}}\widetilde{k_{b+}}[B]+\widetilde{k_{a-}}k_{b-}. \end{aligned}$$

There are two possible solutions of these two equations for $k_{a-},\, k_{b+}$, given by

99 $$\begin{aligned} \begin{pmatrix}k_{a-}\\ k_{b+}\end{pmatrix} =\begin{pmatrix}\widetilde{k_{b+}}[B]+\widetilde{k_{b-}} \\ \frac{\widetilde{k_{a-}}-\widetilde{k_{b-}}}{[B]} \end{pmatrix} \qquad \text {or}\qquad \begin{pmatrix}k_{a-}\\ k_{b+}\end{pmatrix} =\begin{pmatrix}\widetilde{k_{a-}} \\ \widetilde{k_{b+}} \end{pmatrix}. \end{aligned}$$

Hence the system is only locally structurally identifiable, but not globally. This could be circumvented with prior knowledge about the parameters. If we have prior knowledge that $k_{b-}>k_{a-}$, then the first solution in (99) cannot be satisfied since all parameters must be positive. This implies that the only solution that fits within the requirements is the second solution, and hence, that all parameters are identifiable. Remarkably, it is shown in [44] that $k_{b-}>k_{a-}$ if and only if [*AR*](*t*) is monotonic. Therefore, an experimental timecourse readout showing no peak in [*AR*](*t*), together with an equilibrium concentration-response curve for ligand *A* results in identifiability of all five parameters $(k_{a-}, k_{a+}, k_{b-}, k_{b+}, R_{tot})$. For the same combination of experimental data, but with non-monotonic [*AR*](*t*), only $(k_{a+}, k_{b-}, R_{tot})$ are identifiable.

We may also consider an equilibrium concentration-response curve for varying concentration of the competition ligand *B*, as in [44], in addition to the single time course and the concentration-response for [*A*] described above. For example, taking three equilibrium experiments with concentrations ([*A*], [*B*]) = $([A]_{1},[B]_{1})$, $([A]_{2},[B]_{1})$ and $([A]_{1},[B]_{2})$ and corresponding readouts $\phi _{1}$, $\phi _{2}$ and $\phi _{3}$ gives the following system for the equilibrium parameters $K_{A}, K_{B}$ and $R_{tot}$:

100 $$\begin{aligned} \frac{K_A[A]_1R_{tot}}{1+K_A[A]_1+K_B[B]_1}&=\phi _1,\quad \frac{K_A[A]_2R_{tot}}{1+K_A[A]_2+K_B[B]_1}=\phi _2, \nonumber \\ \frac{K_A[A]_1R_{tot}}{1+K_A[A]_1+K_B[B]_2}&=\phi _3. \end{aligned}$$

Solving these for the equilibrium parameters, $K_A$, $K_B$ and $R_{tot}$, gives the unique solution

$$\begin{aligned} R_{tot}&= \frac{\phi _1\phi _2([A]_1-[A]_2)}{[A]_1\phi _2-[A]_2\phi _1}, \\ K_{B}&= \frac{\phi _{2}(\phi _{1}-\phi _{3})([A]_{1}-[A]_{2})}{\phi _{3}[A]_{2}[B]_{2}(\phi _{1}-\phi _{2}) - \phi _{2}[A]_{1}[B]_{1}(\phi _{1}-\phi _{3}) + \phi _{1}[A]_{2}[B]_{1}(\phi _{2}-\phi _{3})}, \\ K_{A}&= \frac{(1+K_B[B])([A]_1\phi _2-[A]_2\phi _1)}{[A]_1[A]_2(\phi _1-\phi _2)}, \end{aligned}$$

and thus determines all three of these parameters to be identifiable. Combining these with time course results, as in (90), we find the model to be globally structurally identifiable.

#### Washout experiments

In this section, we combine the results from an association time course experiment, in (90), with a washout experiment. To determine identifiability using washout experiment data we set $[A]=0$ in equation (36a–c) to simulate the washout of ligand *A* (a similar analysis is possible for washout of ligand *B*, or of both ligands). This results in the reduced model

101a $$[R]^{\prime}= -k_{b+}[B][R]+k_{a-}[AR]+k_{b-}[BR],$$

101b $$[AR]^{\prime}= -k_{a-}[AR],$$

101c $$[BR]^{\prime}= k_{b+}[B][R]-k_{b-}[BR].$$

For this system, the initial conditions are unknown so we assume

101d $$\begin{aligned}{}[R](0)=R_{tot}-[AR]_w-[BR]_w,\qquad [AR](0)=[AR]_w, \qquad [BR](0)=[BR]_w. \end{aligned}$$

The only measured quantity is $[AR]$ and so the output is

101e $$\begin{aligned} y=[AR]. \end{aligned}$$

Assuming that $k_{b-}$ is known, the washout model has unknown parameters ${\textbf {p}}=(k_{a-},k_{b+},R_{tot})$. We again use the Taylor series method. The first Taylor coefficient is given by

102 $$\begin{aligned} y=AR, \qquad \Rightarrow \qquad y_0=[AR]_w, \end{aligned}$$

and the quantity $[AR]_w$ is known. The second coefficient is determined by

103 $$\begin{aligned} y'=AR'=-k_{a-}[AR], \qquad \Rightarrow \qquad y'_0=-k_{a-}[AR]_w, \end{aligned}$$

giving that $k_{a-}$ is identifiable. Further coefficients of the Taylor series give no new information, and so only $k_{a-}$ is identifiable from dissociation data (again, as in Sect. 4.1.2, $R_{tot}$ is not identifiable from washout alone). So use of this washout experiment in conjunction with the binding experiment of Sect. 3.1 allows us to consider $k_{a-}$ as known in (90). We then determine identifiability using the combination of experiments by considering the possible solution, for $(k_{a+},k_{b+},R_{tot})$, of the system

104 $$\begin{aligned} k_{a+}R_{tot}&= \widetilde{k_{a+}}\widetilde{R_{tot}}, \end{aligned}$$

105 $$\begin{aligned} k_{a+}[A]+k_{b+}[B]&= \widetilde{k_{a+}}[A]+\widetilde{k_{b+}}[B], \end{aligned}$$

106 $$\begin{aligned} k_{b-}k_{a+}[A]+k_{a-}k_{b+}[B]&= k_{b-}\widetilde{k_{a+}}[A]+k_{a-}\widetilde{k_{b+}}[B]. \end{aligned}$$

It is straightforward to show that the system has a unique solution $k_{a+}=\widetilde{k_{a+}}$, $k_{b+}=\widetilde{k_{b+}}$, $R_{tot}=\widetilde{R_{tot}}$. We conclude that the Motulsky-Mahan problem, considering the combination of both one binding timecourse and one washout timecourse, is now globally identifiable.

#### Multiple time courses

There are two ways in which we can use multiple sets of time course data to determine identifiability of this model. Taking the coefficients as stated in equation (90) we consider multiple time courses with either several concentrations of *A* or several concentrations of *B*, giving either

107 $$\begin{aligned} \varvec{\zeta }({\textbf {p}})_i&=\begin{bmatrix} k_{a+}R_{tot}\\ k_{a+}[A]_i+k_{b+}[B]+k_{a-}\\ k_{a+}k_{b-}[A]_i+k_{a-}k_{b+}[B]+k_{a-}k_{b-}\end{bmatrix} \nonumber \\ \; \text {or}\quad \varvec{\zeta }({\textbf {p}})_i&=\begin{bmatrix} k_{a+}R_{tot}\\ k_{a+}[A]+k_{b+}[B]_i+k_{a-}\\ k_{a+}k_{b-}[A]+k_{a-}k_{b+}[B]_i+k_{a-}k_{b-}\end{bmatrix} . \end{aligned}$$

for $i=1,2,... \;$. If we study the first case, having time courses for two *A* concentrations, we find the identifiable parameter combinations, in addition to the single identifiable parameter $k_{b-}$, are given by

108 $$\begin{aligned} \varvec{\zeta }({\textbf {p}})=\begin{bmatrix} k_{a+}R_{tot}\\ k_{a+}[A]_1+k_{b+}[B]+k_{a-}\\ k_{a+}k_{b-}[A]_1+k_{a-}k_{b+}[B]+k_{a-}k_{b-}\\ k_{a+}[A]_2+k_{b+}[B]+k_{a-}\\ k_{a+}k_{b-}[A]_2+k_{a-}k_{b+}[B]+k_{a-}k_{b-}\end{bmatrix}. \end{aligned}$$

Solving $\varvec{\zeta }({\textbf {p}})=\varvec{\zeta }(\widetilde{{\textbf {p}}})$, we find that $k_{a+}$ and $R_{tot}$ are identifiable and that $k_{a-}$ and $k_{b+}$ again satisfy (99). The identifiability properties of the Motulsky-Mahan system combining timecourses for two different values of [*A*] are the same as those for the single timecourse plus the concentration-response curve for [*A*].

When we instead consider having time courses for two concentrations of *B*, we obtain the vector of identifiable parameter combinations (in addition to $k_{b-}$, which we know is identifiable from a single timecourse)

109 $$\begin{aligned} \varvec{\zeta }({\textbf {p}})=\begin{bmatrix} k_{a+}R_{tot}\\ k_{a+}[A]+k_{b+}[B]_1+k_{a-}\\ k_{a+}k_{b-}[A]+k_{a-}k_{b+}[B]_1+k_{a-}k_{b-}\\ k_{a+}[A]+k_{b+}[B]_2+k_{a-}\\ k_{a+}k_{b-}[A]+k_{a-}k_{b+}[B]_2+k_{a-}k_{b-}\end{bmatrix}. \end{aligned}$$

Solving $\varvec{\zeta }({\textbf {p}})=\varvec{\zeta }(\widetilde{{\textbf {p}}})$ this time gives that all parameters are identifiable from these two time courses.

### Pre-dimerised G protein-coupled receptor binding

In Sect. 3.2 we explored identifiability for a model of dimeric receptor binding with a single ligand. The schematic for this is given by

$$\begin{array}{*{20}c} {{\text{A}}{\mkern 1mu} \, + {\mkern 1mu} \,{\text{R}}\,{\mkern 1mu} \mathop {\mathop {\, \rightleftharpoons }\limits^{{k_{{a + }} }} }\limits_{{k_{{a - }} }} {\mkern 1mu} \,\,{\text{AR}},\,} & {{\text{A}}\,{\mkern 1mu} + {\mkern 1mu} \,{\text{AR}}\,\,{\mkern 1mu} \mathop {\mathop \rightleftharpoons \limits^{{\alpha _{ + } k_{{b + }} }} }\limits_{{\alpha _{ - } k_{{b - }} }} {\mkern 1mu} \,\,{\text{ARA,}}} \\ \end{array}$$

and the system of equations is given in (61a–e). We found that from a single time course we have no identifiable parameters. Recall that this analysis gave the following identifiable parameter combinations (see (66))

110 $$\begin{aligned} \varvec{\zeta }({\textbf {p}})=\begin{bmatrix} k_{a+}R_{tot}\\ 2\alpha _+k_{a+}[A]+\alpha _-k_{a-}\\ k_{a+}[A]+k_{a-}+\alpha _+k_{a+}[A]+\alpha _-k_{a-}\\ \alpha _+k_{a+}^2[A]^2+\alpha _-k_{a+}k_{a-}[A]+\alpha _-k_{a-}^2 \end{bmatrix}. \end{aligned}$$

#### Saturation curves

We first consider the combination of (110) with information from an equilibrium concentration-response curve which is parameterised by equilibrium parameters, $K_A=k_{a+}/k_{a-}$, $\alpha =\alpha _+/\alpha _-$ and $R_{tot}$. The expression for the concentration of ligand bound at equilibrium, $A_{b}$ was determined in our previous work [55], and is given by

111 $$\begin{aligned} A_{b}=[AR]+2[ARA]=\frac{(K_A[A]+2\alpha K_A^2[A]^2)}{1+K_A[A]+\alpha K_A^2[A]^2}R_{tot}. \end{aligned}$$

Taking three points on the dose-response curve, that is, three different concentrations of $[A]_i$, we have

112 $$\begin{aligned} \frac{(K_A[A]_i+2\alpha K_A^2[A]_i^2)}{1+K_A[A]_i+\alpha K_A^2[A]_i^2}R_{tot}=[A_b]_i,\qquad i=1,2,3, \end{aligned}$$

where $[A_b]_i$ denotes the corresponding measurement for the concentration of ligand $[A]_i$. Equation (112), with $i=1,2,3$, is a system of three equations which can be solved for $K_A$, $\alpha$ and $R_{tot}$. This can be done by using a symbolic equation solver (for example, in MATLAB [40] or Mathematica [58]), and using MATLAB Symbolic Toolbox, we find a unique solution to (112), using just three points. The expressions are extremely lengthy and impractical to write down, and therefore we refrain from doing so here.

We now treat $K_A$, $\alpha$ and $R_{tot}$ as known quantities, together with identifiable parameter combinations (110) to determine identifiability of the parameters in ${\textbf {p}}=(\alpha _+,\alpha _-,k_{a+},k_{a-})$. The first of the equations, after setting $\varvec{\zeta }({\textbf {p}})=\varvec{\zeta }(\widetilde{{\textbf {p}}})$ in (110), yields

113 $$\begin{aligned} k_{a+}R_{tot}=\widetilde{k_{a+}}R_{tot}, \qquad \Rightarrow \qquad k_{a+}=\widetilde{k_{a+}}. \end{aligned}$$

Clearly, $k_{a+}$ is identifiable. Combining this with the known equilibrium parameter $K_A=k_{a+}/k_{a-}$, we find that $k_{a-}$ is also identifiable. The second and third equations from $\varvec{\zeta }({\textbf {p}})=\varvec{\zeta }(\widetilde{{\textbf {p}}})$ can be simplified to

114 $$\begin{aligned} 2\alpha _+k_{a+}[A]+\alpha _-k_{a-}&= 2\widetilde{\alpha _+}k_{a+}[A]+\widetilde{\alpha _-}k_{a-}\end{aligned}$$

115 $$\begin{aligned} \alpha _+k_{a+}[A]+\alpha _-k_{a-}&= \widetilde{\alpha _+}k_{a+}[A]+\widetilde{\alpha _-}k_{a-}. \end{aligned}$$

It is straightforward to show that $\alpha _+=\widetilde{\alpha _+}$ and $\alpha _-=\widetilde{\alpha _-}$, and thus all four parameters $\alpha _+, \alpha _-, k_{a+}$ and $k_{a-}$ (in addition to $R_{tot}$, from the equilibrium analysis) are identifiable.

#### Washout experiments

Here we consider washout experimental data. The corresponding model for washout of the ligand is given by setting $[A]=0$ in equations (61a–e), giving

116a $$\begin{aligned} \frac{d[R]}{dt}&=k_{a-}[AR], \end{aligned}$$

116b $$\begin{aligned} \frac{d[AR]}{dt}&= -k_{a-}[AR]+\alpha _-k_{a-}[ARA], \end{aligned}$$

116c $$\begin{aligned} \frac{d[ARA]}{dt}&=-\alpha _-k_{a-}[ARA]. \end{aligned}$$

The initial conditions are unknown so we assume

116d $$\begin{aligned} R(0)=R_{tot}-[AR]_w-[ARA]_w,\qquad [AR](0)=[AR]_w, \qquad [ARA](0)=[ARA]_w, \end{aligned}$$

where subscript *w* denotes the value at the start of washout, and $[AR]_w$ and $[ARA]_w$ are unknown. The output remains as

116e $$\begin{aligned} y=[AR]+2[ARA]. \end{aligned}$$

We again use the Taylor series method to determine identifiability in this section. Through repeated differentiation of *y* and substitution of the initial conditions we obtain the vector of coefficients as

117 $$\begin{aligned} \varvec{\zeta }_{1}({\textbf {p}})=\begin{bmatrix} [AR]_w+2[ARA]_w \\ -k_{a-}([AR]_w+\alpha _-[ARA]_w) \\ k_{a-}^2([AR]_w+(\alpha _-- 1) \alpha _-[ARA]_w) \\ -k_{a-}^3([AR]_w+(\alpha _-^2 - \alpha _-- 1) \alpha _-[ARA]_w) \\ k_{a-}^4([AR]_w+(\alpha _-^{3}-\alpha _-^2 - \alpha _-- 1) \alpha _-[ARA]_w) \end{bmatrix}. \end{aligned}$$

Since $R_{tot}$ does not appear in $\varvec{\zeta }_{1}$ here, we note that it is not identifiable from the washout model alone, as in Sect. 4.1.2. Further, while the parameters $\alpha _{-}$ and $k_{a-}$ are sought, we have introduced new parameters $[AR]_w$ and $[ARA]_w$, the initial conditions, which are also unknown and are intertwined in the identifiable parameter combinations in (117). Although we do not require them to be identifiable, the analysis requires us to consider them. In Sects. 4.1.2 and 4.2.2, the analysis was simpler given that the new parameter $[AR]_w$ was immediately identifiable as the measured output.

At this point, we may define ${\textbf {p}}=(\alpha _-, k_{a-},[AR]_w,[ARA]_w)$ for the washout experiment and proceed by attempting to solve $\zeta _{1}({\textbf {p}})=\zeta _{1}(\widetilde{{\textbf {p}}})$ to determine the identifiability of individual parameters. Thereafter, we could return to the association timecourse result (110) to determine identifiability of those individual parameters. Given the level of complexity seen in (117), we would use symbolic computation here. Alternatively, our computation could consider ${\textbf {p}}=(\alpha _+,\alpha _-,k_{a+},k_{a-},R_{tot},[AR]_w,[ARA]_w)$ for the two experiments (association and washout) combined. Then the combined identifiable are groupings given by

118 $$\begin{aligned} \varvec{\zeta }_{2}({\textbf {p}})=\begin{bmatrix} k_{a+}R_{tot}\\ 2\alpha _+k_{a+}[A]+\alpha _-k_{a-}\\ k_{a+}[A]+k_{a-}+\alpha _+k_{a+}[A]+\alpha _-k_{a-}\\ \alpha _+k_{a+}^2[A]^2+\alpha _-k_{a+}k_{a-}[A]+\alpha _-k_{a-}^2 \\ [AR]_w+2[ARA]_w \\ -k_{a-}([AR]_w+\alpha _-[ARA]_w) \\ k_{a-}^2([AR]_w+(\alpha _-- 1) \alpha _-[ARA]_w) \\ -k_{a-}^3([AR]_w+(\alpha _-^2 - \alpha _-- 1) \alpha _-[ARA]_w) \\ k_{a-}^4([AR]_w+(\alpha _-^{3}-\alpha _-^2 - \alpha _-- 1) \alpha _-[ARA]_w) \end{bmatrix}. \end{aligned}$$

Now when solving $\varvec{\zeta }_{2}({\textbf {p}})=\varvec{\zeta }_{2}(\widetilde{{\textbf {p}}})$, we find (by symbolic computation, see Appendix 4) the unique solution

119 $$\begin{aligned}&\alpha _+=\widetilde{\alpha _+}, \; \, \alpha _-=\widetilde{\alpha _-}, \; \, k_{a+}=\widetilde{k_{a+}}, \; \, k_{a-}=\widetilde{k_{a-}}, \; \, R_{tot}=\widetilde{R_{tot}}, \nonumber \\&\qquad \qquad [AR]_w=\widetilde{[AR]_w}, \; \, [ARA]_w=\widetilde{[AR]_w}. \end{aligned}$$

Hence we conclude that the combination of association (61a–e) and washout (116a–e) results in all five of the parameters $(\alpha _+,\alpha _-,k_{a+},k_{a-},R_{tot})$ being globally identifiable.

#### Multiple experiments

Next, we consider association timecourse data obtained from experiments each with a different ligand concentration [*A*]. We aim to determine the minimum number of concentrations needed to ensure full identifiability. Each of these experiments yields identifiable parameter combinations, as obtained in (110), with $[A]_i$ as the concentration for experiment $y_i$. This gives the identifiable parameter combinations

120 $$\begin{aligned} \varvec{\zeta }({\textbf {p}})_i=\begin{bmatrix} k_{a+}R_{tot}\\ k_{a+}[A]_iR_{tot}(2\alpha _+k_{a+}[A]_i+\alpha _-k_{a-}) \\ k_{a+}[A]_i+k_{a-}+\alpha _+k_{a+}[A]_i+\alpha _-k_{a-}\\ \alpha _+k_{a+}^2[A]_i^2+\alpha _-k_{a+}k_{a-}[A]_i+\alpha _-k_{a-}^2 \end{bmatrix}. \end{aligned}$$

Upon assuming data for two experiments, we choose $i=1, 2$ to give the vector of identifiable combinations as

121 $$\begin{aligned} \varvec{\zeta }({\textbf {p}})=\begin{bmatrix} k_{a+}R_{tot}\\ 2\alpha _+k_{a+}[A]_1+\alpha _-k_{a-}\\ k_{a+}[A]_1+k_{a-}+\alpha _+k_{a+}[A]_1+\alpha _-k_{a-}\\ \alpha _+k_{a+}^2[A]_1^2+\alpha _-k_{a+}k_{a-}[A]_1+\alpha _-k_{a-}^2 \\ 2\alpha _+k_{a+}[A]_2+\alpha _-k_{a-}\\ k_{a+}[A]_2+k_{a-}+\alpha _+k_{a+}[A]_2+\alpha _-k_{a-}\\ \alpha _+k_{a+}^2[A]_2^2+\alpha _-k_{a+}k_{a-}[A]_2+\alpha _-k_{a-}^2 \end{bmatrix}. \end{aligned}$$

To determine identifiability we again set $\varvec{\zeta }({\textbf {p}})=\varvec{\zeta }(\widetilde{{\textbf {p}}})$ and solve for ${\textbf {p}}=(\alpha _+,\alpha _-,k_{a+},k_{a-},R_{tot})$. It is a matter of simple algebraic manipulation to find that

122 $$\begin{aligned} \alpha _+=\widetilde{\alpha _+}, \quad \alpha _-=\widetilde{\alpha _-}, \quad k_{a+}=\widetilde{k_{a+}}, \quad k_{a-}=\widetilde{k_{a-}}, \quad R_{tot}=\widetilde{R_{tot}}. \end{aligned}$$

So we conclude that only two data sets are required to ensure that the system is globally structurally identifiable.

## Discussion

Structural identifiability analysis (SIA) is an often-overlooked element of modelling of biological systems [12]. The notion of identifiability is well known and appreciated, but in practice the complexity of the calculations that are required to draw conclusions regarding the identifiability of a given ODE system is often a barrier. While the “classical” SIA methods of transfer function, Taylor Series and similarity transformation have been applied to a number of pharmacokinetics models in the literature, SIA is largely absent from receptor theory and analytical pharmacology studies. Here, we have introduced SIA methodology to receptor theory via application of these three classical methods to three widely adopted ligand-receptor binding schematics of biological importance. Our analysis has yielded new identifiability results for single-timecourse receptor theory outputs, plus a significant and crucial focus on approaches to mitigating non-identifiability via the addition of further experiments. In addition, the article provides a pedagogical, tutorial-style introduction to formal identifiability analysis, aligned with the aim of bringing SIA to a broader audience [7].

Our key results include a formal SIA verification that, for the model of ligand *A* binding monomeric receptor, the quantities $k_{a+}R_{tot}$ and $k_{obs}=k_{a+}[A]+k_{a-}$ (the so-called *observed on-rate* [34]) are globally identifiable, see expression (6). For this monomeric receptor model, we also confirm mathematically the intuitively known fact that all kinetic parameters and the receptor concentration become identifiable when adding a washout experiment, an equilibrium saturation binding model or simply when using timecourses for two ligand concentrations.

For the Motulsky-Mahan competition binding model (36a–e), it was already known that if the total receptor $R_{tot}$ and labelled ligand constants $k_{a+}, k_{a-}$ are known, then the unlabelled ligand constants $k_{b+}$ and $k_{b-}$ are theoretically identifiable from a single [*AR*] timecourse [15, 44]. Our SIA (see expressions (43, 44)) shows that $k_{b-}$ is in fact identifiable without the need for a priori knowledge of any constants. Furthermore, if $k_{a+}$ is known, then $R_{tot}$ is also identifiable without knowledge of any other parameters, or if $R_{tot}$ is known, then $k_{a+}$ is identifiable. From these results, it is clear that a single timecourse may yield more practical, quantitative parametric information than previously thought. In addition, we have shown that SIA enables a formal strategy for constructing an identifiable system when also considering washout experiments and/or multiple ligand concentrations (Sect. 4.2). Recent computational studies of the Motulsky-Mahan model have focused on questions of practical identifiability and parameter estimation [18, 49] and have noted a relationship between binding timescales and estimation reliability. Further investigation into this relationship will benefit from the analytical results and methods presented in the current work.

We have also shown new results from SIA applied to a model of GPCR dimers in Sect. 3.2. This model has been previously used with experimental data for total bound ligand to partially quantify the important effect of cooperativity (reporting an equilibrium parameter) across a dimer [41] without discussion of parameter identifiability properties. Our new analysis indicates that no model parameters are identifiable from a single binding timecourse. However, when using multiple ligand concentrations, all kinetic parameters and the total receptor concentration are identifiable. These results that were so far unknown provide both practical guides for the estimation of kinetic cooperativity and an extension of the recent theoretical study of cooperativity and dimer binding dynamics given in [55].

The models we have considered have been low-dimensional (at most third-order) and linear, which is typical of many ligand-binding models in receptor theory. For such models, the implementation of the three classical SIA methods is tractable. Given the relative conceptual simplicity of these approaches compared to more recent methods developed for larger biology and systems biology models [3, 12, 46, 48], the introduction of SIA to receptor theory via these three methods has been shown to be viable, although we remark that the Taylor Series approach is cumbersome in some cases, benefiting from symbolic computation tools. The transfer function method is relatively straightforward, and reflects earlier use of the Laplace Transform in textbook PK parameter estimation discussion [19]. The Taylor Series approach and a modified similarity transformation method suitable for nonlinear models [10, 50] are potentially suitable for second-order nonlinear binding models such as those arising in ligand-induced receptor dimerisation [56], and simple nonlinear models for receptor-mediated cell responses via kinetic operational models of agonism [26]. To bridge the gap to higher-dimensional models of interest in receptor theory (including binding of allosteric modulators [24], more detailed operational models [26] and G protein activation [59]), recent novel algorithmic and computational approaches including Exact Arithmetic Rank [46], input–output method [3, 31] and singular value decomposition of sensitivity matrices [48] appear to be promising methods.

We conclude by proposing the following studies as future work.

1. Apply SIA to a binding model for ligand-induced receptor dimerisation. Recent analysis of this nonlinear ODE model has shed new light on dynamic cooperativity effects across the dimers, and the model has been validated by fitting it to real timecourse data [56]. SIA is required to determine the theoretical identifiability of the model parameters (in preparation [57]).
2. Apply an identifiability analysis to the Motulsky-Mahan model combining both the structural identifiability results presented in the current work and the recent practical identifiability and estimation results in [15, 18, 49] to derive an overall strategy for informing parameter estimation studies for this widely-used model.
3. Perform a bridging-the-gap analysis which implements the Exact Arithmetic Rank [46], input–output method [3, 31] and singular value decomposition method [48] to the models in the current work, the ligand-induced dimerisation model and kinetic operational models, to compare ease of implementation and computational cost.

### Supplementary Information

Below is the link to the electronic supplementary material.

Supplementary file1 (PDF 49 KB)

## Appendix 1 Similarity transformation method for competition binding model

In this appendix, we apply the similarity transform method, as introduced in Sect. 2.3, to the Motulsky-Mahan, competition binding system. To apply this method we require the system to be controllable and observable. In the full system (36a–e) we have $G = (0, 0, 0)^T$, and so the controllability matrix is

123 $$\begin{aligned} \mathcal {C}=\begin{bmatrix} 0 &{} \vdots &{} 0 &{} \vdots &{} 0 \\ 0 &{} \vdots &{} 0 &{} \vdots &{} 0 \\ 0 &{} \vdots &{} 0 &{} \vdots &{} 0 \end{bmatrix}, \end{aligned}$$

so we are unable to determine controllability. Therefore, we instead reformulate and use the reduced form, as in (37a, b) in Sect. 3.1.1. Recall that in this form we define

124 $$\begin{aligned}&{\textbf {x}}=\begin{bmatrix} [AR]\\ [BR]\end{bmatrix}, \quad F=\begin{bmatrix} -(k_{a+}[A]+k_{a-}) &{} -k_{a+}[A]\\ -k_{b+}[B]&{} -(k_{b+}[B]+k_{b-}) \end{bmatrix}, \nonumber \\&G=\begin{bmatrix} k_{a+}[A]R_{tot}\\ k_{b+}[B]R_{tot}\end{bmatrix},\quad H=\begin{bmatrix} 1&0 \end{bmatrix}. \end{aligned}$$

In this case, the dimension is $n=2$, and the controllability matrix is given by

125 $$\begin{aligned} \mathcal {C}=\begin{bmatrix} k_{a+}[A]R_{tot}&{} -k_{a+}[A]R_{tot}(k_{a+}[A]+k_{a-}+k_{b+}[B]) \\ k_{b+}[B]R_{tot}&{} -k_{b+}[B]R_{tot}(k_{a+}[A]+k_{b+}[B]+k_{b-}) \end{bmatrix}, \end{aligned}$$

which has $rank(\mathcal {C})=2$, and so the system is controllable. The observability matrix is found to be

126 $$\begin{aligned} \mathcal {O}=\begin{bmatrix} 1 &{} 0 \\ -(k_{a+}[A]+k_{a-}) &{} -k_{a+}[A]\end{bmatrix}, \end{aligned}$$

which also has $rank(\mathcal {O})=2$, therefore, both controllability and observability conditions are met.

Next, we assume that there exists a transformation matrix

127 $$\begin{aligned} T=\begin{bmatrix} t_{11} &{} t_{12} \\ t_{21} &{} t_{22} \end{bmatrix}, \end{aligned}$$

and assume that conditions (32a–e) have to hold. Then, step by step, we draw conclusions from these conditions, and find the entries in matrix *T*. We first note that (32b) is satisfied for any *T* as ${\textbf {x}}_0 = \tilde{{\textbf {x}}}_0 = (0,0)^T$. We now check condition (32e), which becomes

128 $$\begin{aligned} \begin{bmatrix} 1&0 \end{bmatrix}=\begin{bmatrix} 1&0 \end{bmatrix} \begin{bmatrix} t_{11} &{} t_{12} \\ t_{21} &{} t_{22} \end{bmatrix} \quad \text {and hence}\quad t_{11}=1, \; t_{12}=0. \end{aligned}$$

Then, condition (32d) implies that

129 $$\begin{aligned} \begin{bmatrix} 1 &{} 0 \\ t_{21} &{} t_{22} \end{bmatrix}\begin{bmatrix} \widetilde{k_{a+}}[A]\widetilde{R_{tot}} \\ \widetilde{k_{b+}}[B]\widetilde{R_{tot}} \end{bmatrix}=\begin{bmatrix} k_{a+}[A]R_{tot}\\ k_{b+}[B]R_{tot}\end{bmatrix}, \end{aligned}$$

which leads to

130 $$\begin{aligned} \widetilde{k_{a+}}[A]\widetilde{R_{tot}}&=k_{a+}[A]R_{tot}, \end{aligned}$$

131 $$\begin{aligned} t_{21}\widetilde{k_{a+}}[A]\widetilde{R_{tot}}+t_{22}\widetilde{k_{b+}}[B]\widetilde{R_{tot}}&=k_{b+}[B]R_{tot}. \end{aligned}$$

The first of these equations, since $[A]$ is known, yields

132 $$\begin{aligned} \widetilde{k_{a+}}\widetilde{R_{tot}}=k_{a+}R_{tot}, \end{aligned}$$

which is the first identifiable parameter combination. Next, (131) can be written as

133 $$\begin{aligned} t_{22}=\frac{k_{b+}[B]R_{tot}-t_{21}\widetilde{k_{a+}}[A]\widetilde{R_{tot}}}{\widetilde{k_{b+}}[B]\widetilde{R_{tot}}}. \end{aligned}$$

So far, we have found that

134 $$\begin{aligned} T=\begin{bmatrix} 1 &{} 0 \\ t_{21} &{} \dfrac{k_{b+}[B]R_{tot}-t_{21}\widetilde{k_{a+}}[A]\widetilde{R_{tot}}}{\widetilde{k_{b+}}[B]\widetilde{R_{tot}}} \end{bmatrix}. \end{aligned}$$

Next, for condition (32c) to hold

135 $$\begin{aligned} T\begin{bmatrix} -(\widetilde{k_{a+}}[A]+\widetilde{k_{a-}}) &{} -\widetilde{k_{a+}}[A]\\ -\widetilde{k_{b+}}[B]&{} -(\widetilde{k_{b+}}[B]+\widetilde{k_{b-}}) \end{bmatrix} =\begin{bmatrix} -(k_{a+}[A]+k_{a-}) &{} -k_{a+}[A]\\ -k_{b+}[B]&{} -(k_{b+}[B]+k_{b-}) \end{bmatrix}T, \end{aligned}$$

needs to be satisfied. We multiply both sides by $-1$, and write (135) as

136 $$\begin{aligned} \begin{bmatrix} M_{11} &{} M_{12} \\ M_{21} &{} M_{22} \end{bmatrix}=\begin{bmatrix} N_{11} &{} N_{12} \\ N_{21} &{} N_{22} \end{bmatrix}, \end{aligned}$$

where

$$\begin{aligned} M_{11}&=\widetilde{k_{a+}}[A]+\widetilde{k_{a-}}, \\ M_{12}&=\widetilde{k_{a+}}[A], \\ M_{21}&=t_{21}\widetilde{k_{a-}}+\dfrac{k_{b+}[B]R_{tot}}{\widetilde{R_{tot}}},\\ M_{22}&=\dfrac{\widetilde{k_{b+}}k_{b+}[B]^2\widetilde{R_{tot}}+k_{b+}\widetilde{k_{b-}}[B]R_{tot}-t_{21}\widetilde{k_{a+}}\widetilde{k_{b-}}[A]\widetilde{R_{tot}}}{\widetilde{k_{b+}}[B]\widetilde{R_{tot}}}, \end{aligned}$$

and

$$\begin{aligned} N_{11}&=k_{a+}[A]+k_{a-}+t_{21}k_{a+}[A], \\ N_{12}&=\dfrac{k_{a+}[A](k_{b+}[B]R_{tot}-t_{21}\widetilde{k_{a+}}[A]\widetilde{R_{tot}})}{\widetilde{k_{b+}}[B]\widetilde{R_{tot}}}, \\ N_{21}&=k_{b+}[B]+t_{21}k_{b+}[B]+t_{21}k_{b-}, \\ N_{22}&=\dfrac{(k_{b+}[B]+k_{b-})(k_{b+}[B]R_{tot}-t_{21}\widetilde{k_{a+}}[A]\widetilde{R_{tot}})}{\widetilde{k_{b+}}[B]\widetilde{R_{tot}}}. \end{aligned}$$

We now equate the entries of matrices *M* and *N*. First we solve $M_{12}=N_{12}$ for $t_{21}$, giving

137 $$\begin{aligned} \widetilde{k_{a+}}[A]&=\frac{k_{a+}[A](k_{b+}[B]R_{tot}-t_{21}\widetilde{k_{a+}}[A]\widetilde{R_{tot}})}{\widetilde{k_{b+}}[B]\widetilde{R_{tot}}}, \nonumber \\ t_{21}&=\frac{k_{a+}k_{b+}[B]R_{tot}-\widetilde{k_{a+}}\widetilde{k_{b+}}[B]\widetilde{R_{tot}}}{k_{a+}\widetilde{k_{a+}}[A]\widetilde{R_{tot}}}. \end{aligned}$$

We equate $M_{11}=N_{11}$, which together with the above expression for $t_{21}$, becomes

138 $$\begin{aligned} \widetilde{k_{a+}}[A]+\widetilde{k_{a-}}=\frac{k_{a+}k_{b+}[B]R_{tot}+\widetilde{k_{a+}}k_{a-}\widetilde{R_{tot}}+k_{a+}\widetilde{k_{a+}}[A]\widetilde{R_{tot}}-\widetilde{k_{a+}}\widetilde{k_{b+}}[B]\widetilde{R_{tot}}}{\widetilde{k_{a+}}\widetilde{R_{tot}}}. \end{aligned}$$

Since we have already determined that $\widetilde{k_{a+}}\widetilde{R_{tot}}=k_{a+}R_{tot}$ (see (132)), this results in

139 $$\begin{aligned} \widetilde{k_{a+}}[A]+\widetilde{k_{a-}}=\frac{\widetilde{k_{a+}}k_{a-}\widetilde{R_{tot}}+k_{a+}\widetilde{k_{a+}}[A]\widetilde{R_{tot}}+\widetilde{k_{a+}}k_{b+}[B]\widetilde{R_{tot}}-\widetilde{k_{a+}}\widetilde{k_{b+}}[B]\widetilde{R_{tot}}}{\widetilde{k_{a+}}\widetilde{R_{tot}}}. \end{aligned}$$

Hence we find

140 $$\begin{aligned} \widetilde{k_{a+}}[A]+\widetilde{k_{a-}}+\widetilde{k_{b+}}[B]=k_{a+}[A]+k_{a-}+k_{b+}[B], \end{aligned}$$

which is the second identifiable combination. Equating $M_{22}=N_{22}$ gives

141 $$\begin{aligned} \frac{k_{a+}k_{b+}[B]R_{tot}+\widetilde{k_{a+}}\widetilde{k_{b-}}\widetilde{R_{tot}}}{k_{a+}\widetilde{R_{tot}}}=\frac{\widetilde{k_{a+}}(k_{b+}[B]+k_{b-})}{k_{a+}}. \end{aligned}$$

With $\widetilde{k_{a+}}\widetilde{R_{tot}}=k_{a+}R_{tot}$, this can simplified to

142 $$\begin{aligned} \widetilde{k_{b-}}=k_{b-}, \end{aligned}$$

confirming that the parameter $k_{b-}$ is identifiable. Finally, equating $M_{21}=N_{21}$, gives, after some simplification (again using MATLAB Symbolic Toolbox)

143 $$\begin{aligned} \widetilde{k_{b+}}(\widetilde{k_{a-}}-\widetilde{k_{b-}})=k_{b+}(k_{a-}-k_{b-}), \end{aligned}$$

which gives a fourth identifiable parameter combination.

We note that the transformation matrix is given by

144 $$\begin{aligned} T=\begin{bmatrix} 1 &{} 0 \\ \dfrac{k_{a+}k_{b+}[B]R_{tot}-\widetilde{k_{a+}}\widetilde{k_{b+}}[B]\widetilde{R_{tot}}}{k_{a+}\widetilde{k_{a+}}[A]\widetilde{R_{tot}}} &{} \dfrac{\widetilde{k_{a+}}}{k_{a+}} \end{bmatrix}, \end{aligned}$$

which has determinant

145 $$\begin{aligned} \det (T)=\dfrac{\widetilde{k_{a+}}}{k_{a+}}. \end{aligned}$$

As $\det (T)\ne 0$, we find that condition (32a) holds.

In summary we have $k_{b-}$ as identifiable, and identifiable parameter combinations

146 $$\begin{aligned} \varvec{\zeta }({\textbf {p}})=\begin{bmatrix} k_{a+}R_{tot}\\ k_{b+}(k_{a-}-k_{b-}) \\ k_{a+}[A]+k_{b+}[B]+k_{a-}\end{bmatrix}. \end{aligned}$$

That is, the same parameter combinations are found to be identifiable as for the previous methods (see (44) and (59)).

## Appendix 2 Taylor series approach to GPCR homodimer model

Here, we apply the Taylor Series method to the GPCR dimer model. In (73a–d), we established that for the GPCR homodimer model, a vector of identifiable combinations is then given by

147a $$\begin{aligned} \varvec{\zeta }_{1}({\textbf {p}})=\begin{bmatrix} k_{a+}R_{tot}\\ P \\ Q \\ R \end{bmatrix}, \end{aligned}$$

where

147b $$\begin{aligned} P&= \alpha _+k_{a+}[A]-k_{a+}[A]-k_{a-}, \end{aligned}$$

147c $$\begin{aligned} Q&= k_{a+}^2[A]^2-\alpha _+^2k_{a+}^2[A]^2-\alpha _+k_{a+}^2[A]^2+2k_{a+}k_{a-}[A]-\alpha _-k_{a+}k_{a-}[A]+k_{a-}^2, \end{aligned}$$

147d $$\begin{aligned} R&= \alpha _+^3k_{a+}^3[A]^2+\alpha _+^2k_{a+}^3[A]^3+\alpha _+k_{a+}^3[A]^3-k_{a+}^3[A]^3 \nonumber \\&\qquad + 2\alpha _+^2\alpha _-k_{a+}^2k_{a-}[A]^2+\alpha _+\alpha _-k_{a+}^2k_{a-}[A]^2 \nonumber \\&\qquad +\alpha _+^2k_{a+}^2k_{a-}[A]^2-3k_{a+}^2k_{a-}[A]^2 \nonumber \\&\qquad +\alpha _+\alpha _-^2k_{a+}k_{a-}^2[A]-\alpha _+k_{a+}k_{a-}^2[A]-3k_{a+}k_{a-}^2[A]-k_{a-}^3. \end{aligned}$$

By ad hoc algebraic manipulations, largely using MATLAB Symbolic Toolbox, we find that

147e $$\begin{aligned} \frac{2PQ-P^{3}-R}{Q-P^{2}}&= 2\alpha _+k_{a+}[A]+\alpha _-k_{a-}, \end{aligned}$$

147f $$\begin{aligned} \frac{PQ-R}{Q-P^{2}}&= k_{a+}[A]+k_{a-}+\alpha _+k_{a+}[A]+\alpha _-k_{a-}, \end{aligned}$$

147g $$\begin{aligned} \frac{PR-Q^{2}}{Q-P^{2}}&= \alpha _+k_{a+}^2[A]^2+\alpha _-k_{a+}k_{a-}[A]+\alpha _-k_{a-}^2, \end{aligned}$$

showing that the combinations in (74) are recoverable from (147a–g) and hence identifiable. A sample MATLAB code is given in Supplementary Materials.

## Appendix 3 Similarity transformation method for GPCR dimer model

In this appendix, we apply the similarity transformation method to the GPCR dimer model. Recall that the reduced form of the system is given by (9) where

148 $$\begin{aligned} F&=\begin{bmatrix} -(k_{a+}[A]+k_{a-}+\alpha _+k_{a+}[A]) &{} \alpha _-k_{a-}-k_{a+}[A]\\ \alpha _+k_{a+}[A]&{} -\alpha _-k_{a-}\end{bmatrix}, \nonumber \\&\qquad G=\begin{bmatrix} k_{a+}[A]R_{tot}\\ 0 \end{bmatrix},\qquad H=\begin{bmatrix} 1&2 \end{bmatrix}. \end{aligned}$$

Before we determine identifiability for the system, we first check whether the controllability and observability conditions are satisfied. Using (29) the controllability matrix for this system is found to be

149 $$\begin{aligned} \mathcal {C}=\begin{bmatrix} k_{a+}[A]R_{tot}&{} -k_{a+}[A]R_{tot}(k_{a+}[A]+k_{a-}+\alpha _+k_{a+}[A]) \\ 0 &{} \alpha _+k_{a+}^2[A]^2R_{tot}\end{bmatrix}, \end{aligned}$$

and the observability matrix is found, using (30), to be

150 $$\begin{aligned} \mathcal {O}=\begin{bmatrix} 1 &{} 2 \\ \alpha _+k_{a+}[A]-k_{a+}[A]-k_{a-}&{} -(\alpha _-k_{a-}+k_{a+}[A])\end{bmatrix}. \end{aligned}$$

As $\textrm{rank}(\mathcal {C})=\textrm{rank}(\mathcal {O})=2$ we conclude that the system is both controllable and observable, hence we continue with the identifiability analysis.

Assuming the existence of a linear transformation matrix

151 $$\begin{aligned} T=\begin{bmatrix} t_{11} &{} t_{12} \\ t_{21} &{} t_{22} \end{bmatrix}, \end{aligned}$$

and that conditions (32a–e) hold, we begin to draw conclusions. We first note that ${\textbf {x}}(0)={\textbf {0}}$ implies that condition (32b) automatically holds. We now apply condition (32e), giving

152 $$\begin{aligned} \begin{bmatrix} 1&2 \end{bmatrix}=\begin{bmatrix} 1&2 \end{bmatrix}\begin{bmatrix} t_{11} &{} t_{12} \\ t_{21} &{} t_{22} \end{bmatrix}. \end{aligned}$$

From this we determine

153 $$\begin{aligned} t_{11}&=1-2t_{21}, \end{aligned}$$

154 $$\begin{aligned} t_{12}&=2-2t_{22}. \end{aligned}$$

We next apply condition (32d), giving

155 $$\begin{aligned} \begin{bmatrix} 1-2t_{21} &{} 2-2t_{22} \\ t_{21} &{} t_{22}\end{bmatrix} \begin{bmatrix} \widetilde{k_{a+}}[A]\widetilde{R_{tot}} \\ 0 \end{bmatrix}=\begin{bmatrix} k_{a+}[A]R_{tot}\\ 0 \end{bmatrix}. \end{aligned}$$

From the bottom row, we find

156 $$\begin{aligned} t_{21}=0. \end{aligned}$$

Then the top row gives the first identifiable parameter combination as

157 $$\begin{aligned} \widetilde{k_{a+}}\widetilde{R_{tot}}=k_{a+}R_{tot}. \end{aligned}$$

With (156) the transformation matrix now becomes

158 $$\begin{aligned} T=\begin{bmatrix} 1 &{} 2-2t_{22} \\ 0 &{} t_{22} \end{bmatrix}. \end{aligned}$$

Applying condition (32c) and determining the left-hand and right-hand side we obtain

159 $$\begin{aligned} \begin{bmatrix} M_{11} &{} M_{12} \\ M_{21} &{} M_{22} \end{bmatrix}=\begin{bmatrix} N_{11} &{} N_{12} \\ N_{21} &{} N_{22} \end{bmatrix}, \end{aligned}$$

where

$$\begin{aligned} M_{11}&=\widetilde{\alpha _+}\widetilde{k_{a+}}[A]-\widetilde{k_{a+}}[A]-\widetilde{k_{a-}}-2t_{22}\widetilde{\alpha _+}\widetilde{k_{a+}}[A],\\ M_{12}&=2t_{22}\widetilde{\alpha _-}\widetilde{k_{a-}}-\widetilde{k_{a+}}[A]-\widetilde{\alpha _-}\widetilde{k_{a-}}, \\ M_{21}&=t_{22}\widetilde{\alpha _+}\widetilde{k_{a+}}[A], \\ M_{22}&=-t_{22}\widetilde{\alpha _-}\widetilde{k_{a-}}, \end{aligned}$$

and

$$\begin{aligned} N_{11}&=-(k_{a+}[A]+k_{a-}+\alpha _+k_{a+}[A]), \\ N_{12}&=t_{22}(k_{a+}[A]+2k_{a-}+2\alpha _+k_{a+}[A]+\alpha _-k_{a-})-2(k_{a+}[A]+k_{a-}+\alpha _+k_{a+}[A]),\\ N_{21}&=\alpha _+k_{a+}[A], \\ N_{22}&=2\alpha _+k_{a+}[A]-2t_{22}\alpha _+k_{a+}[A]-t_{22}\alpha _-k_{a-}. \end{aligned}$$

We solve $M_{21}=N_{21}$ for $t_{22}$, which gives

160 $$\begin{aligned} t_{22}=\frac{\alpha _+k_{a+}}{\widetilde{\alpha _+}\widetilde{k_{a+}}}. \end{aligned}$$

We note that, while it is possible to begin with a different matrix entry, and therefore have a different expression for $t_{22}$, this will still give the same identifiability results. We substitute the expression for $t_{22}$ into the remaining matrix entries. Solving $M_{11}=N_{11}$, we find, after some simplification

161 $$\begin{aligned} \widetilde{k_{a+}}[A]+\widetilde{k_{a-}}-\widetilde{\alpha _+}\widetilde{k_{a+}}[A]=k_{a+}[A]+k_{a-}-\alpha _+k_{a+}[A]. \end{aligned}$$

Equating $M_{22}=N_{22}$ and $M_{12}=N_{12}$, we obtain

162 $$\begin{aligned} 2\widetilde{\alpha _+}\widetilde{k_{a+}}[A]+\widetilde{\alpha _-}\widetilde{k_{a-}}=2\alpha _+k_{a+}[A]+\alpha _-k_{a-}, \end{aligned}$$

and

163 $$\begin{aligned} \widetilde{\alpha _+}\widetilde{k_{a+}}^2[A]^2+\widetilde{\alpha _-}\widetilde{k_{a+}}\widetilde{k_{a-}}[A]+\widetilde{\alpha _-}\widetilde{k_{a-}}^2=\alpha _+k_{a+}^2[A]^2+\alpha _-k_{a+}k_{a-}[A]+\alpha _-k_{a-}^2, \end{aligned}$$

respectively. Hence, we find the identifiable parameter combinations for this model given in the vector

164 $$\begin{aligned} \varvec{\zeta }_{2}({\textbf {p}})=\begin{bmatrix} k_{a+}R_{tot}\\ 2\alpha _+k_{a+}[A]+\alpha _-k_{a-}\\ \alpha _+k_{a+}^2[A]^2+\alpha _-k_{a+}k_{a-}[A]+\alpha _-k_{a-}^2 \\ k_{a+}[A]+k_{a-}-\alpha _+k_{a+}[A]\end{bmatrix}. \end{aligned}$$

The transformation matrix is given by

165 $$\begin{aligned} T=\begin{bmatrix} 1 &{} 2-2\dfrac{\alpha _+k_{a+}}{\widetilde{\alpha _+}\widetilde{k_{a+}}} \\ 0 &{} \dfrac{\alpha _+k_{a+}}{\widetilde{\alpha _+}\widetilde{k_{a+}}} \end{bmatrix}, \end{aligned}$$

which has determinant

166 $$\begin{aligned} \det (T)=\frac{\alpha _+k_{a+}}{\widetilde{\alpha _+}\widetilde{k_{a+}}}\ne 0, \end{aligned}$$

and so we confirm that condition (32a) holds.

Note that the combinations listed in (164) and (74) are not identical. However, since the second and fourth entries in (164) sum to give $(k_{a+}[A]+k_{a-}+\alpha _+k_{a+}[A]+\alpha _-k_{a-})$, we conclude that the identifiable parameter combinations are the same as those in (66) and (74), namely

167 $$\begin{aligned} \varvec{\zeta }({\textbf {p}})=\begin{bmatrix} k_{a+}R_{tot}\\ 2\alpha _+k_{a+}[A]+\alpha _-k_{a-}\\ k_{a+}[A]+k_{a-}+\alpha _+k_{a+}[A]+\alpha _-k_{a-}\\ \alpha _+k_{a+}^2[A]^2+\alpha _-k_{a+}k_{a-}[A]+\alpha _-k_{a-}^2 \end{bmatrix}. \end{aligned}$$

## Appendix 4 Combined association and washout computation for GPCR homodimer model

In Supplementary Materials, we include a MATLAB code which, when run after the code for the Appendix 2 computations, returns a unique solution to the equation $\varvec{\zeta }_{2}({\textbf {p}})=\varvec{\zeta }_{2}(\widetilde{{\textbf {p}}})$ for ${\textbf {p}}=(\alpha _+,\alpha _-,k_{a+},k_{a-},R_{tot},[AR]_w,[ARA]_w)$ and $\varvec{\zeta }_{2}$ given by (118).
