# Correction to: Classical structural identifiability methodology applied to low-dimensional dynamic systems in receptor theory

**DOI:** 10.1007/s10928-023-09879-3

**Published:** 2023-07-26

**Authors:** Carla White, Vivi Rottschäfer, Lloyd Bridge

**Affiliations:** 1 Swansea University, Swansea, UK; 2 Leiden University, Leiden, The Netherlands; 3 University of Amsterdam, Amsterdam, The Netherlands; 4 University of the West of England, Bristol, UK

**Correction to: Journal of Pharmacokinetics and Pharmacodynamics**

10.1007/s10928-023-09870-y

In this article, the equation 39 did not display correctly. The equation 39 has been set correctly now.

The original article has been corrected.

