# Supplementary material for: Classical structural identifiability methodology applied to low-dimensional dynamic systems in receptor theory
Source: J Pharmacokinet Pharmacodyn. 2023 Jun 30;51(1):39–63. doi: 10.1007/s10928-023-09870-y (PMC10884104; doi:10.1007/s10928-023-09870-y)
Supplement: Supplementary file 1 — Supplementary file1 (PDF 49 KB) [file 10928_2023_9870_MOESM1_ESM.pdf]

Classical structural identifiability methodology applied to low-dimensional dynamic systems in receptor theory

Supplementary materials

Carla White[1], Vivi Rottschäfer[2,3] and Lloyd Bridge[4*]

[1] Swansea University, Swansea, UK.
[2] Leiden University, Leiden, Netherlands.
[3] University of Amsterdam, Amsterdam, Netherlands.
[4] University of the West of England, Bristol, UK. * Corresponding author.

Contents

1 MATLAB code for Taylor Series approach to GPCR homodimer model 1

2 MATLAB code for Taylor Series for GPCR homodimer model with washout 2

1 MATLAB code for Taylor Series approach to GPCR homodimer model (see Appendix B of main text)

A sample MATLAB code for the Taylor Series calculations for the GPCR homodimer model (see Appendix B of the main text) is given below.

```
% Appendix B MATLAB code
%------------------------
clear; close all; clc
syms k_ap k_am a_p a_m A R_t R(t) AR(t) ARA(t)
% system equations for full association system
f=[-k_ap*A*R+k_am*AR;k_ap*A*R-k_am*AR-a_p*k_ap*A*AR+a_m*k_am*ARA;
a_p*k_ap*A*AR-a_m*k_am*ARA];
y=AR+2*ARA;
x=[R;AR;ARA];
x0=[R_t;0;0];
n=length(x0);
% calculate first Taylor coefficient
y0(1,1)=subs(y,x,x0); %note that this is zero

% calculate all remaining coefficients
for i=2:2*n-1
    [y,y0_new]=TaylorSeriesSI(f,y,x,x0);
    y0(i,1) = y0_new;
end

simplify(y0) %note a factor f y0(2) = A*Rt*kap in all terms
%so B1,B2,B3,B4 are identifiable
B1 = y0(2);
B2 = y0(3)/y0(2);
B3 = y0(4)/y0(2);
B4 = y0(5)/y0(2);
simplify([B1;B2;B3;B4])

P = B2;
Q = B3;
R = B4;
```

```
%note that C1,C2,C3,C4 will be identifiable
C1 = B1;
C2 = (2*P*Q - P^3 - R)/(Q - P^2);
C3 = (P*Q-R)/(Q-P^2);
C4 = (P*R-Q^2)/(Q-P^2);
simplify([C1;C2;C3;C4])

function [y_new, y0] = TaylorSeriesSI(sys,y,x,x0)
% This function takes inputs of sys, y, x and x0. It differentiates y,
% substitutes in the ODEs (this is the new y vector) and then the initial conditions
% (the Taylor coefficients). The new y and Taylor coeffs are returned.
yd = diff(y);
y_new = subs(yd,diff(x),sys);
y0 = subs(y_new,x,x0);
end
```

2 MATLAB code for combined association and washout computation for GPCR homodimer model (see Appendix D of main text)

Here, we include a MATLAB code which, when run after the code in Section 1 of this supplementary document, returns a unique solution to the equation $\boldsymbol{\zeta}_2(\mathbf{p}) = \boldsymbol{\zeta}_2(\widetilde{\mathbf{p}})$ for

$$\mathbf{p} = (\alpha_+, \alpha_-, k_{a+}, k_{a-}, R_{tot}, [AR]_w, [ARA]_w), \tag{2.1}$$

and $\boldsymbol{\zeta}_2$ given by:

$$\boldsymbol{\zeta}_2(\mathbf{p}) = \begin{bmatrix} k_{a+}R_{tot} \\ 2\alpha_+ k_{a+}[A] + \alpha_- k_{a-} \\ k_{a+}[A] + k_{a-} + \alpha_+ k_{a+}[A] + \alpha_- k_{a-} \\ \alpha_+ k_{a+}^2 [A]^2 + \alpha_- k_{a+} k_{a-}[A] + \alpha_- k_{a-}^2 \\ [AR]_w + 2[ARA]_w \\ -k_{a-}([AR]_w + \alpha_-[ARA]_w) \\ k_{a-}^2([AR]_w + (\alpha_- - 1)\alpha_-[ARA]_w) \\ -k_{a-}^3([AR]_w + (\alpha_-^2 - \alpha_- - 1)\alpha_-[ARA]_w) \\ k_{a-}^4([AR]_w + (\alpha_-^3 - \alpha_-^2 - \alpha_- - 1)\alpha_-[ARA]_w) \end{bmatrix}. \tag{2.2}$$

```
% Appendix D MATLAB code
%------------------------

y0_assoc = [C1;C2;C3;C4];

% define washout system with A=0
syms k_ap k_am a_p a_m A R_t R(t) AR(t) ARA(t) AR_w ARA_w
A=0;
f=[-k_ap*A*R+k_am*AR;k_ap*A*R-k_am*AR-a_p*k_ap*A*AR+a_m*k_am*ARA;
a_p*k_ap*A*AR-a_m*k_am*ARA];

y=AR+2*ARA;
x=[R;AR;ARA];
x0=[R_t-AR_w-ARA_w;AR_w;ARA_w];
n=length(x0);
% calculate forst Taylor coefficient
y0(1,1)=subs(y,x,x0);
% calculate all remaining coefficients
```

```
for i=2:2*n-1
    [y,y0_new]=TaylorSeriesSI(f,y,x,x0);
    y0(i,1) = y0_new;
end
% resulting identifiable combinations, that is z(p) for the washout sys
y0_dis=simplify(y0)

% solving washout and time course eqns together
syms k_ap1 k_am1 a_p1 a_m1 R_t1 AR_w1 ARA_w1

combs = [y0_assoc;y0_dis];
combs_alt = subs(combs,[k_ap,k_am,a_p,a_m,R_t,AR_w,ARA_w],...
    [k_ap1,k_am1,a_p1,a_m1,R_t1,AR_w1,ARA_w1]);
eq2 = combs==combs_alt;
S = solve(eq2,[k_ap,k_am,a_p,a_m,R_t,AR_w,ARA_w], 'Real', true)
```
